# Gut microbial production of lithocholic acid reprograms pro-resolutive macrophages to enhance vedolizumab responsiveness via the TGR5/FXR–NF-κB axis

**DOI:** 10.1093/ismejo/wrag028

**Published:** 2026-02-16

**Authors:** Bing Han, Hongtao Wen, Ya Li, Yucai Wang, Xiaoping Lv, Mei Kang, Wei Huang, Yining Lan, Shilin Tong, Mengying Zhang, Deyi Chen, Chen Zhu, Yong Jiang, Daiyuan Tang

**Affiliations:** Department of Gastroenterology, The First Affiliated Hospital of Zhengzhou University, Zhengzhou 450052, China; Centre for Leading Medicine and Advanced Technologies of IHM, The First Affiliated Hospital of University of Science and Technology of China, Hefei, 230036, China; National Key Laboratory of Immune Response and Immunotherapy, University of Science and Technology of China, Hefei, 230026, China; Department of Gastroenterology, The First Affiliated Hospital of Zhengzhou University, Zhengzhou 450052, China; Department of Gastroenterology, The First Affiliated Hospital of Zhengzhou University, Zhengzhou 450052, China; National Key Laboratory of Immune Response and Immunotherapy, University of Science and Technology of China, Hefei, 230026, China; Department of Gastroenterology, The First Affiliated Hospital of Guangxi Medical University, Nanning, 530021, China; Department of Gastroenterology, The First Affiliated Hospital of Zhengzhou University, Zhengzhou 450052, China; Department of Gastroenterology, The First Affiliated Hospital of Guangxi Medical University, Nanning, 530021, China; Department of Neurology, The First Affiliated Hospital of Guangxi Medical University, Nanning, 530021, China; Department of Clinical Immunology, The First Affiliated Hospital of Zhengzhou University, Zhengzhou, 450052, China; Department of Clinical Immunology, The First Affiliated Hospital of Zhengzhou University, Zhengzhou, 450052, China; Department of Gastroenterology, The First People’s Hospital of Qinzhou, Qinzhou, 535009, China; Centre for Leading Medicine and Advanced Technologies of IHM, The First Affiliated Hospital of University of Science and Technology of China, Hefei, 230036, China; Department of Clinical Immunology, The First Affiliated Hospital of Zhengzhou University, Zhengzhou, 450052, China; Institute of Infection and Immunity, Henan Academy of Innovations in Medical Science, Zhengzhou, 450003, China; Department of Gastroenterology, The First Affiliated Hospital of Zhengzhou University, Zhengzhou 450052, China; Centre for Leading Medicine and Advanced Technologies of IHM, The First Affiliated Hospital of University of Science and Technology of China, Hefei, 230036, China; National Key Laboratory of Immune Response and Immunotherapy, University of Science and Technology of China, Hefei, 230026, China

**Keywords:** gut microbiota, bile acid metabolites, lithocholic acid, immune response, biomarker, Crohn’s disease

## Abstract

Crohn’s disease (CD) is a complex chronic transmural inflammatory bowel disease. Although vedolizumab (VDZ) markedly improves clinical outcomes in CD, treatment non-response remains a significant limitation, constraining its broader utility. Elucidating the mechanisms underlying VDZ responsiveness is thus critically needed. In this research, we employed a humanized mouse model of 2,4,6-trinitrobenzene sulfonic acid–induced colitis to investigate VDZ treatment response in CD. Our findings indicate that VDZ significantly alleviated disease phenotypes in a portion of CD mice. Integrated metagenomic and metabolomic profiling identified baseline gut microbiota–derived secondary bile acids as potential predictors of VDZ efficacy. Subsequent fecal microbiota transplantation from clinical donors into pseudo-germ-free mice confirmed that gut microbial composition critically influences VDZ responsiveness. Targeted metabolomics further pinpointed lithocholic acid (LCA) as a key microbially derived metabolite correlated with therapeutic remission. Single-cell RNA sequencing also revealed that intestinal macrophages serve as pivotal mediators of LCA-driven modulation of treatment outcomes. Furthermore, transcriptomic analyses demonstrated that LCA polarizes macrophages toward an M2-resolutive phenotype via concurrent engagement of the *TGR5*/*FXR* and their downstream nuclear factor kappa-B (NF-κB) pathways. Ultimately, using a conditioned medium co-culture system, we established that the regulatory effects of pro-resolutive macrophage niche on treatment response in a manner dependent on the *TGR5/FXR*–NF-κB axis. Taken together, our study elucidates a microbiota-immune circuit in which gut microbial metabolite LCA augments VDZ responsiveness in CD by reprogramming macrophages toward a pro-resolutive phenotype via the *TGR5/FXR*–NF-κB signaling network. These insights provide a mechanistic foundation for biomarker development and personalized therapeutic strategies in inflammatory bowel disease.

## Graphical Abstract

**Graphical Abstract:**
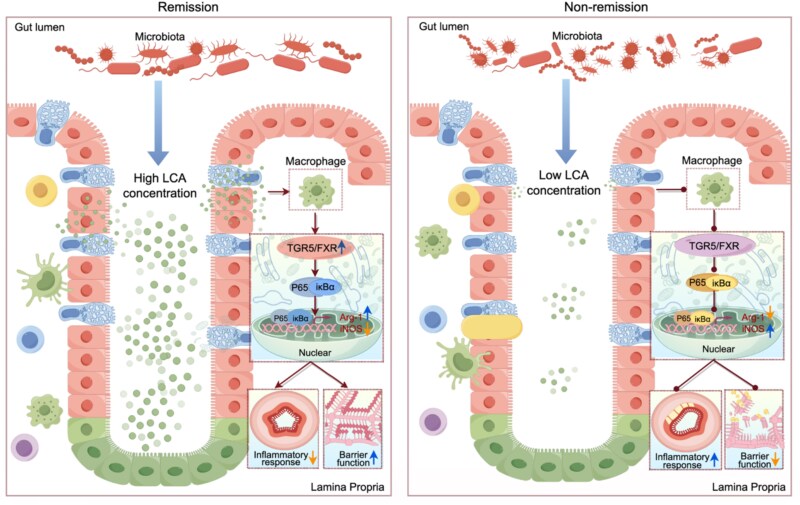

## Introduction

Crohn’s disease (CD) is a chronic inflammatory disorder that primarily targets the gastrointestinal tract. It is distinguished by a pattern of symptoms that can reoccur intermittently, leading to episodes of flare-ups followed by periods of remission [1]. This complex condition is the result of a combination of various factors, including genetic predispositions, environmental triggers, and the composition of the gut microbiome. Together, these elements contribute to an abnormal immune response within the mucosal lining of the intestines, which in turn results in a disruption of the integrity of the epithelial barrier that protects the gut [2]. Modern therapeutic approaches prioritize attaining deep and enduring remission to avert complications and interrupt the disease’s progressive trajectory. Over the last 30 years, treatments for CD have seen remarkable progress. Until recently, anti–tumor necrosis factor drugs served as the cornerstone of treatment. A novel category of therapies, such as anti-α4β7-integrin, has emerged to inhibit leukocytes from migrating to the intestinal mucosa [3].

Vedolizumab (VDZ), a gut-selective anti-α4β7-integrin monoclonal antibody, represents a pivotal treatment option for inducing and maintaining remission in CD [4, 5]. Nevertheless, primary non-response remains a significant clinical challenge specifically in CD [6]. Despite this, patients with CD demonstrate ongoing improvement over a span of 2 years, emphasizing the significance of identifying individuals likely to respond positively to VDZ before it is stopped [7]. The saturation level of the α4β7 receptor does not always align with the therapeutic outcomes observed from VDZ therapy. This observation suggests that although the blockade of α4β7 receptors is a significant aspect of VDZ’s functionality, it does not fully account for the underlying mechanisms by which VDZ exerts its effects [8, 9]. Therefore, other pathways or factors may also play a pivotal role in the drug’s efficacy, indicating a more complex interaction that goes beyond mere receptor blockade. A recent study discovered that in CD patients treated with VDZ, the populations of macrophages and the expression of pattern recognition receptors changed significantly, both of which are important components of the intestinal mucosal innate immune system [10]. As a result, our study concentrated on the development of innovative biomarkers and associated mechanisms to forecast treatment outcomes in CD.

Patients with CD have intestinal microecological imbalance, marked by a reduction of anti-inflammatory bacteria [11]. Approximately a third of individuals diagnosed with CD showcase a significantly elevated presence of mucosa-associated adherent-invasive *Escherichia coli*. These bacteria can penetrate the protective mucosal barrier, adhere to and invade the intestinal cell lining, and survive and proliferate within macrophages [12, 13]. Moreover, individuals diagnosed with CD exhibit a decrease in the population of the anti-inflammatory *Faecalibacterium prausnitzii* [14, 15]. Recently, a study involving patients with CD who began biological agent treatment revealed that patients in remission exhibited greater community α-diversity while showing reduced β-diversity in comparison to those not in remission [16]. Therefore, variations in baseline gut microbiota abundance are linked to responses to treatment. Nevertheless, the ways in which particular microbes and the minor molecules they influence interact to forecast the reaction to VDZ in CD are still not fully understood.

Changes in the gut microbiota among patients with CD have been associated with variations in metabolites, particularly bile acids (BAs) [17–20]. BAs represent a distinctive chemical characteristic of steroids that primarily arise from cholesterol metabolism [21]. Gut microbiota transform primary BAs into secondary BAs during the digestive process, clearly indicating that intestinal dysbiosis associated with CD mainly affects gut microbiota and the metabolism of BAs. In reality, metabolomic investigations of CD patients have revealed altered microbial BAs in feces, especially increased conjugated primary BA levels and decreased secondary BA levels [17–20]. In a colitis mouse model, co-supplementation with long *Bifidobacteria* enhances the effectiveness of infliximab for colitis treatment by raising the levels of secondary BAs like goose deoxycholate and lithocholic acid (LCA) [21]. Especially, LCA is involved in modulating inflammation and immunity [22]. Additionally, various receptors, including *TGR5* and *FXR*, are the focus of attention and are activated by secondary BAs. Most receptors that respond to BAs are widely distributed throughout the digestive system, and their expression is affected by gut microbiota, which undergoes significant changes during colitis [23]. Animals that do not possess the membrane receptor *TGR5* or the nuclear receptor *FXR* exhibit a greater susceptibility to colitis, suggesting that receptors activated by BAs are crucial for maintaining intestinal homeostasis [24, 25]. Recent studies further demonstrate that the stimulation of *TGR5* by BAs can encourage intestinal epithelial cell regeneration, which is necessary for repairing the mucosal barrier after any type of disturbance [23]. Other than that, the activation of *FXR* with synthetic agonists shielded mice from chemically caused intestinal inflammation by inhibiting pro-inflammatory cytokines synthesis and halting goblet cell degeneration [25]. Consequently, activating the *TGR5/FXR* signaling pathways via BAs can help reduce the clinical manifestations of CD.

To bridge this critical knowledge gap, we designed an integrated study employing a humanized mouse model, multi-omics profiling, and functional validations. Our goal was to determine whether specific gut microbial metabolites, particularly secondary bile acids, could influence VDZ responsiveness by reprogramming intestinal macrophages, and to elucidate the underlying molecular circuitry.

## Materials and methods

### Humanized immune system mouse model

In our study, we employed a mouse model with a humanized immune system, as VDZ specifically targets immunological cells derived from humans. At present, it is believed that the hu-CD34^+^ HSC model is one of the most suitable animal models for studying immunotherapy, and it can effectively establish the human innate immune system and lymphocytes [26, 27]. To reconstruct their immune system, 8–10-week-old female NOD.Cg-*Prkdc^scid^Il2rg^em1Smoc^* mice were administered human CD34^+^ hematopoietic stem cells (Biocytogen, Beijing, China). This particular model [huHSC-(M-NSG)] incorporates human immune cells that have been implanted [28, 29]. CD45 is a specific surface antigen of human leukocytes. The content of human CD45^+^ cells in recipient mice is an important indicator characterizing the transplantation effect of human hematopoietic stem/progenitor cells in mice. If the percentage of human CD45^+^ cells exceeds 25%, it indicates that the immune system reconstruction is successful [30, 31]. The proportion of differentiated human CD45^+^ cells in the peripheral blood of mice was detected by flow cytometry. All mice included in the study exceeded the 25% hCD45^+^ threshold (Fig. S1A–D). The α4β7 integrin was also consistently and robustly expressed on human CD45^+^ cells (Fig. S2A–D). That is, the humanized mouse model of the immune system was successfully constructed. Following the confirmation of successful engraftment, the mice were subsequently relocated to our facility for further observation and experimentation. Upon their arrival, mice were housed under specific pathogen-free conditions in individually ventilated cages, with a 12-h light/dark cycle, ambient temperature maintained at 22 ± 1°C, humidity at 55 ± 10%, and provided with autoclaved chow and water *ad libitum*. The experiments were conducted following the established Animal Experiment Guidelines set forth by Zhengzhou University (Approval Number: ZZU-LAC20250912).

### Animal experimentation procedures

The animal experimentation involved four main procedures, which consisted of the following:

1. To evaluate the response to VDZ in CD, humanized mice were randomly assigned to three different groups: control (*n* = 8), model (*n* = 8), and those treated with VDZ (*n* = 16). Based on the published research, VDZ (Cat #28713; Takeda Pharmaceutical, Tokyo, Japan) exhibited a clinically delayed response, thus 50 mg/kg of VDZ was administered intraperitoneally 2 days prior to the 2,4,6-trinitrobenzene sulfonic acid (TNBS) induction, with a further 30 mg/kg of VDZ given on days 0 and 4, respectively [32]. The control and model mice were given equal amounts of PBS. Finally, mice with colitis that were treated with VDZ were classified into two separate groups: one showing remission and the other not showing remission. This categorization was based on noticeable enhancements in several aspects, such as pathological symptoms, the degree of intestinal inflammation, and the performance of the intestinal barrier. The designations remission and non-remission in this preclinical model specifically refer to post-treatment groups stratified by the composite colitis severity endpoint defined herein and are distinct from clinical remission criteria in patients. Stool samples were collected from the subjects at baseline for the purpose of conducting mNGS and non-targeted metabolomics analysis prior to the initiation of treatment with VDZ. Following the treatment period, isopentane was used to euthanize the mice, and samples were subsequently collected on the seventh day for further analysis.
2. To assess the role of gut microbiota in the response to VDZ in CD, we created a humanized gut microbiota using FMT in pseudo-germ-free (PGF) mice. Mice designated as PGF were generated using a recognized protocol from earlier research that incorporates a complex of antibiotics (ABX) [33]. This antibiotic cocktail includes vancomycin (Cat #1404-93-9) at a concentration of 0.5 g/l, ampicillin (Cat #69-52-3) at 1 g/l, metronidazole (Cat #443-48-1) at 1 g/l, and neomycin sulfate (Cat #1405-10-3) at 1 g/l. The four antibiotics mentioned above were sourced from Sigma-Aldrich. Stool samples were taken prior to and following the administration of ABX for 16S rRNA sequencing. To ensure the effective elimination of the majority of gut microbiota in the recipient mice, a group of 8 mice per category was subjected to ABX for a duration of 7 days. Following this antibiotic regimen, the mice were induced with TNBS to provoke an inflammatory response. Concurrently, they received daily transplants of baseline gut microbiota obtained from CD patients, specifically categorized into three groups: those in remission at week 14 (FMT-remission), those not achieving remission by the same time frame (FMT-non-remission), and the baseline microbiota from CD patients (FMT-model). In addition, healthy human feces were utilized as controls (FMT-control). This transplantation process was carried out over the span of 15 consecutive days. After FMT, stool samples from the recipient mice were gathered for 16S rRNA sequencing and targeted metabolomic analysis of BAs.
3. To confirm that the gut microbial metabolite LCA can regulate the therapeutic response level of VDZ, we administered LCA and VDZ simultaneously to CD mice. A total of four distinct groups of humanized mice were created for this study, with each group consisting of 8 mice. The groups include a control group, a model group, a group receiving VDZ treatment, and a group undergoing combined treatment with VDZ and LCA. LCA was obtained from Sigma-Aldrich (Cat #L6250; purity >95%). Based on previous studies, LCA (0.8 mmol/kg) dissolved in corn oil was given via oral gavage to the VDZ + LCA groups from day −2 to day 7 once a day [34, 35], while the remaining three groups received corn oil alone. The administration of VDZ was detailed in the experimental procedure described in Part I. Body weight, disease activity index (DAI), as well as other clinical signs were monitored daily. At day 7, mice were euthanized using isopentane overdose. Colon tissue was used for single-cell sequencing and subsequent analysis.
4. To further demonstrate that intestinal macrophages are important mediators of LCA in regulating VDZ treatment response in CD, we use clodronate liposomes (CLO) to eliminate macrophages in mice as previously described [36]. Mice were randomly assigned to four experimental groups (*n* = 12 per group): VDZ, VDZ + CLO, VDZ + LCA, and VDZ + LCA + CLO. A starting dose of 200 μl CLO (Cat #F70101C; FormuMax Scientific, Sunnyvale, USA) for a mouse body weight of 20–25 g was intraperitoneally injected into mice 2 days before TNBS-induced inflammation. To prevent repopulation of macrophages, repeated injections of 100 μl CLO were performed every 2 days after the first injection until day 4. Control mice received the same dose of PBS liposomes at the same time points. Depletion efficiency was validated on day 4 by flow cytometry (FC) of F4/80^+^ cells in colonic tissue using 4 mice per group, with the remaining 8 mice per group retained for phenotypic assessment.

### Statistical analysis

Data are presented as mean ± standard deviation (SD). Statistical tests were chosen based on data distribution (assessed for normality and homogeneity of variances) and experimental design. Comparisons between two independent groups used the unpaired two-tailed Student’s *t*-test. Comparisons among more than two groups used one-way ANOVA (with Tukey’s *post hoc* test). For high-dimensional omics data, *P*-values were adjusted using the Benjamini–Hochberg false discovery rate correction. For pre-specified hypothesis testing, a threshold of *P* <.05 was applied. All analyses accommodated unequal group sizes through the use of Welch’s correction or nonparametric tests. Analyses were performed in R (v4.3.2) using DESeq2, vegan, and MetaboAnalystR packages, and in GraphPad Prism (v10.0) for basic statistics and graphing.

### Experimental methods

More detailed information regarding the experimental methods used in this study is provided in the supplementary materials, including Tables S1 and S2.

## Results

### VDZ shows significant potential in alleviating the disease phenotypes observed in CD mice

**Figure 1:**
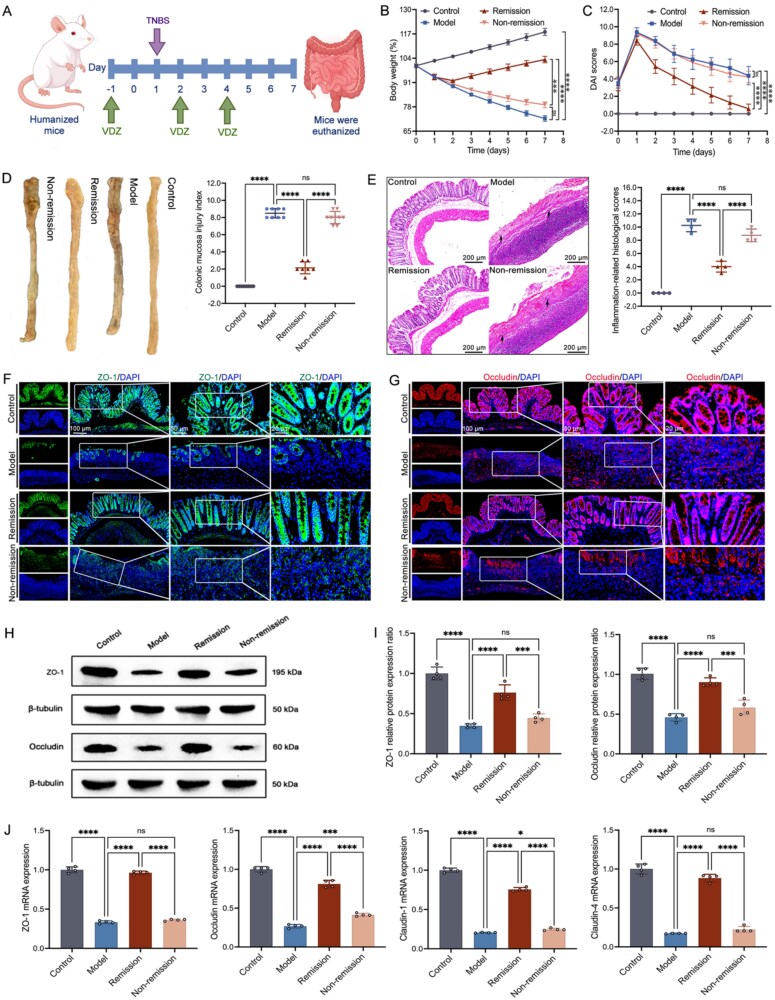
Pathological symptoms mitigated with vedolizumab (VDZ) in CD mice. (A) Schematic diagram of TNBS-induced inflammation and VDZ intervention experiment; (B) body weight change over time (*n* = 7–9); (C) disease activity index (DAI) over time (*n* = 7–9); (D) illustrative images alongside scores related to colonic mucosal damage injury (*n* = 7–9); (E) illustrative images with a scale bar of 200 μm and associated scores of H&E (inflammatory cell infiltration indicated by black arrows, *n* = 4); (F and G) images representative of scale bars measuring 100, 50, and 20 μm show the immunofluorescence of *ZO-1* and *occludin* (*n* = 4); (H and I) representative immunoblots along with the protein levels of *ZO-1* and *occludin* (*n* = 4); (J) real-time quantitative polymerase chain reaction (RT-qPCR) of intestinal barrier function (*n* = 4). Results are expressed as mean ± SD. A one-way analysis of variance was utilized. ^ns^*P* > .05, ^*^P < .05, ^***^P < .001, and ^****^P < .0001.

The colitis triggered by TNBS can effectively mimic the characteristic clinical manifestations of CD [37, 38]. We used TNBS to induce inflammation and administered VDZ intervention (Fig. 1A). Following the administration of TNBS, the mice developed significant weight loss (Fig. 1B). These significant elevations in the DAI and CMDI scores were also observed (Fig. 1C and D). The histological examination of the CD mice demonstrated a range of significant pathological alterations in the mucosal lining of the intestines. Among these findings were extensive areas of ulceration, which signify severe damage to the tissue integrity. There was also a marked infiltration of inflammatory cells, indicating an active immune response to inflammation. Furthermore, the study observed the destruction of crypts, which are essential structures for maintaining the health and function of the intestinal lining. Lastly, there was degradation of the surface epithelium, underscoring the extent of tissue damage and compromising the overall barrier function of the mucosa. Following the administration of VDZ, the remission group showed a considerable enhancement in the previously referenced symptoms (Fig. 1E).

To evaluate the function of the intestinal barrier, we conducted multifaceted tests on tight junction proteins (ZO-1 and occludin). The use of immunofluorescence staining techniques showed a significant reduction in the levels of ZO-1 and occludin within the membranes of intestinal epithelial cells in mice affected by colitis. In contrast to the other groups, the remission group demonstrated a significant increase in the expression of ZO-1 and occludin within the spinous and granular layers of the mucosal tissue (Fig. 1F and G). This observation was further corroborated by WB analysis, which similarly highlighted the enhanced levels of these proteins (Fig. 1H and I). Moreover, compared with the non-remission mice, the mRNA levels of ZO-1 and occludin were significantly higher in the remission mice (Fig. 1J).

**Figure 2:**
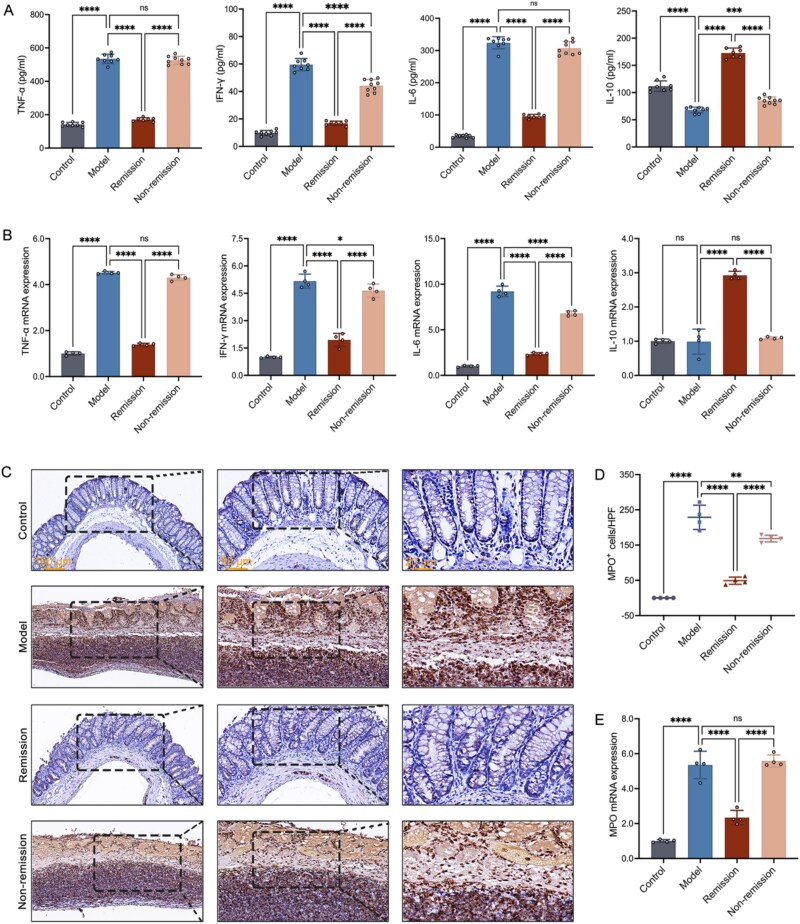
VDZ alleviates intestinal inflammation in CD mice. (A) Serum levels of inflammatory cytokines (*n* = 7–9); (B) RT-qPCR of inflammatory cytokines (*n* = 4); (C) representative images at scale bars of 100, 50, and 20 μm display MPO immunohistochemistry (*n* = 4); (D) quantification of (C) (*n* = 4); (E) colonic MPO expression (*n* = 4). Results are expressed as mean ± SD. A one-way analysis of variance was utilized. ^ns^*P* > .05, ^*^P < .05, ^**^P < .01, ^***^P < .001, and ^****^P < .0001.

The CD mice that were in remission exhibited considerably reduced serum levels of pro-inflammatory cytokines in comparison to those with non-remission. In contrast, the colitis mice that achieved remission exhibited significantly higher concentrations of the anti-inflammatory cytokine (Fig. 2A and B). Additionally, the extent of inflammatory cell infiltration can be assessed by evaluating the levels of MPO. A clear linear relationship has been established between the activity of MPO in the colon and the infiltration of neutrophils in inflamed colonic tissues [39]. During our study, we observed that the MPO levels in mice with colitis were significantly higher when compared to those in control mice, indicating an enhanced inflammatory response. In the group of mice that experienced remission, there was a marked decrease in MPO levels, suggesting that a reduction in MPO may be associated with improved inflammatory conditions (Fig. 2C–E). However, a portion of humanized mice suffering from colitis did not demonstrate any improvement in these disease characteristics despite receiving treatment with VDZ. In other terms, not every mouse with colitis responded positively to VDZ.

### Baseline microbiome profiles show potential for forecasting response to VDZ in CD

**Figure 3:**
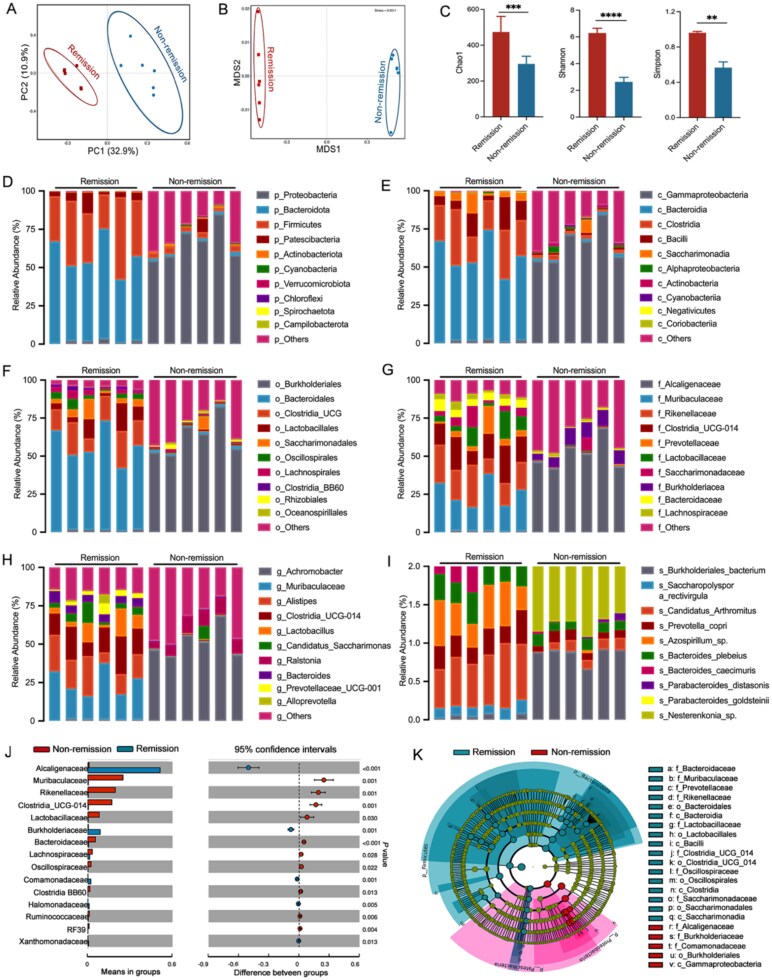
Gut microbiota composition at baseline in CD. (A) Principal component analysis (PCA) score plot visually represents the relationships within the microbiota data; (B) non-metric multidimensional scaling plot further illustrates the differences in microbial composition among individuals; (C) the assessment of community α-diversity, measured through indices such as Chao1, Shannon, and Simpson, provides comparative insights into the microbial diversity within the two studied groups; (D–I) the composition of gut microbiota was analyzed across all levels of classification; (J) the abundance of gut microbiota significantly varied at the genus level between the groups in and out of remission; (K) the abundance of gut microbiota demonstrated significant variability across all classification levels between the groups (*n* = 6 in each group). Results are expressed as mean ± SD. Student’s *t*-test was applied. ^**^P < .01, ^***^P < .001, and ^****^P < .0001.

Due to the recognition of gut dysbiosis as a contributing factor in CD, we employed metagenomic techniques to explore how the gut microbiota influences treatment responses. In the TNBS-induced colitis model, the principal component analysis demonstrated significant differences in operational taxonomic units between the remission group and the non-remission group (Fig. 3A). The non-metric multidimensional scaling plot further reinforced this observation, as it clearly illustrated a significant separation of the OTUs in the groups (Fig. 3B). The findings regarding α-diversity indicate a pronounced disparity in microbial abundance between the remission group and the non-remission group (Fig. 3C). This suggests that the state of remission is associated with distinct microbial community characteristics. Furthermore, when examining the composition of gut microbiota, it was found that there were unique differences among each group at every taxonomic level (Fig. S3A–F), especially between the remission group and the non-remission group (Fig. 3D–I). In detail, there was a high abundance of *Muribaculaceae*, *Rikenellaceae*, *Lactobacillaceae*, *Clostridia*, *Bacteroidaceae*, and *Oscillospiraceae* in these remission-achieving subjects. In contrast, the bacterial families *Alcaligenaceae*, *Burkholderiaceae*, and *Comamonadaceae* showed a marked increase in the mice that did not achieve remission (Fig. 3J and K). Overall, our findings suggest that leveraging baseline microbiome profiles show potential for forecasting response to VDZ.

### Baseline modulation of secondary BA metabolism engage in therapeutic efficacy of VDZ in CD

**Figure 4:**
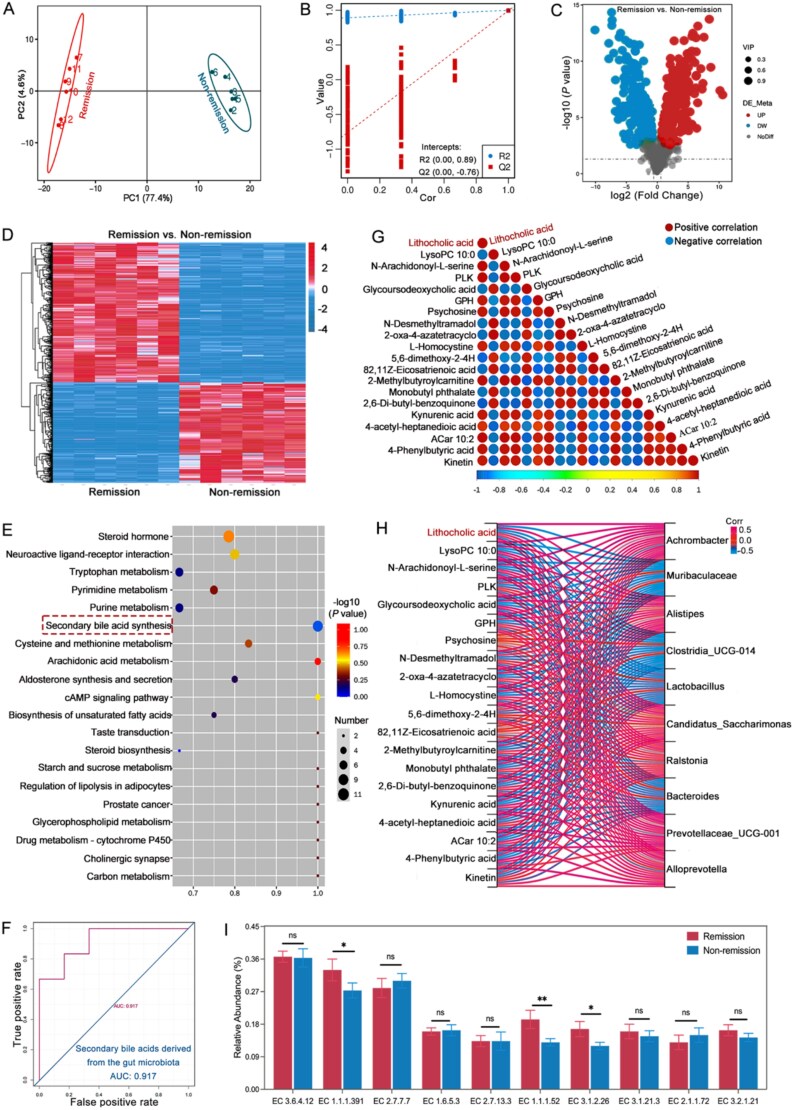
Modulation of bile acids (BAs) at baseline determines treatment response. (A) PCA score plot allows for the identification of patterns in BA metabolism related to treatment response; (B) sort verification plot ensures that the selected features accurately correspond to the respective responses observed in the study; (C) volcano plot effectively highlights the differentially expressed metabolites (upregulated metabolites = 287, downregulated metabolites = 206); (D) clustering heatmap offers a comprehensive overview of the relationships between samples; (E) functional enrichment analysis of metabolites; (F) heatmap displays correlations among significantly altered metabolites from panel (C) (only correlations with *r* > 0.8 and *P* < .01 are shown); (G) association analysis of differential microbiota and differential metabolites; (H) the predictive model showcases the area under the curve derived from the analysis of gut microbiota and its relationship with secondary BA metabolism; (I) comparative analysis of the abundance of enzymes associated with gut microbiota in groups (*n* = 6 in each group). Results are expressed as mean ± SD. Student’s *t*-test was applied. ^ns^*P* > .05, ^*^P < .05, ^**^P < .01.

Considering the important function of metabolism in maintaining gut homeostasis, especially regarding CD, we investigated how serum metabolites might affect responses to treatment. This section used the TNBS-induced colitis model. The hierarchical clustering analysis was performed on all metabolites identified in the groups. Significant differences were observed in the metabolic expression between these groups, suggesting the existence of unique metabolic mechanisms or cellular pathways (Fig. 4A and B). Volcano plots and heatmaps further showed that there are significant differences between the two groups, each with unique differential metabolites (Fig. 4C and D). Further analysis of the metabolites demonstrated that secondary BA metabolism played an important role (Fig. 4E and Fig. S4A). Especially among the differential metabolites, LCA is classified as a secondary BA. LCA shows a direct synergistic effect with most differential metabolites (Fig. 4F). Essentially, metabolites of BA could influence the responses to treatment.

### Baseline metagenomics and metabonomics are integrated to construct a comprehensive response prediction model

A previous study on CD receiving VDZ treatment indicated that the baseline clinical characteristics alone did not adequately predict remission at week 14. Nevertheless, the inclusion of microbial taxa enhanced the ability to forecast outcomes [16]. To further improve the predictive ability, we created an extensive prediction model that included both BA metabolites and gut microbiota. A significant correlation was observed between the gut microbiota–mediated secondary BA metabolites and treatment responses (Fig. 4G). Specifically, initial data comprising gut microbiota and secondary BAs were adequate for forecasting remission (AUC 0.917) (Fig. 4H). Moreover, the group that achieved remission demonstrated a significant increase in the activity of three specific enzymes: EC 1.1.1.391, EC 1.1.1.52, and EC 3.1.2.26 (Fig. 4I and Fig. S4B–D). These enzymes play a crucial role in the biosynthesis of secondary BAs, indicating a significant biochemical change associated with the remission status. This enrichment suggests a potentially critical pathway that may contribute to the therapeutic outcomes observed in the remission group. Consequently, changes in BA metabolism mediated by the gut microbiota may influence responses to treatment.

### Response of CD to VDZ is impacted by reshaping humanized gut microbiota

**Figure 5:**
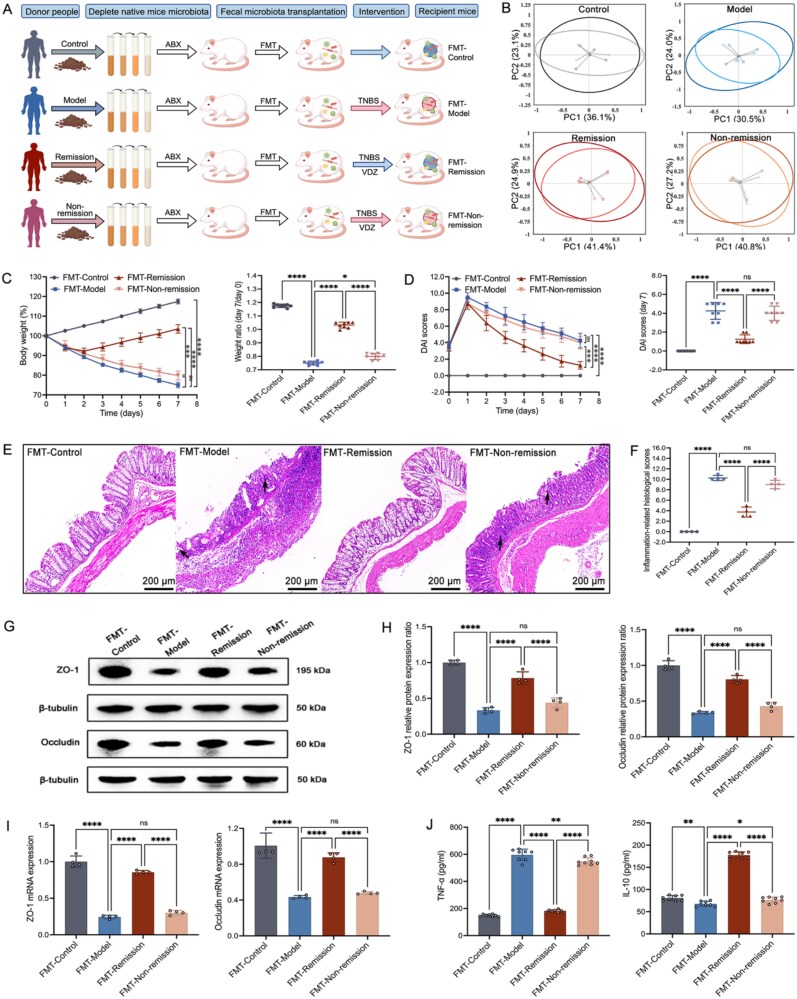
VDZ treatment response impacted by targeting gut microbiota. (A) Schematic illustration of fecal microbiota transplantation to colitis mice; (B) PCA score plot represents the relationships within the microbiota data (*n* = 3); (C) body weight change and day 7 relative weight (*n* = 8); (D) DAI over time and on day 7 (*n* = 8); (E and F) illustrative images with a scale bar of 200 μm and associated scores of H&E (inflammatory cell infiltration indicated by black arrows, *n* = 4); (G and H) illustrative immunoblots along with the protein levels of *ZO-1* and *occludin* (*n* = 4); (I) tight junctions’ mRNA levels (*n* = 4); (J) serum levels of inflammatory cytokines (*n* = 8). Results are expressed as mean ± SD. The one-way analysis of variance and Student’s *t*-test were applied. ^ns^*P* > .05, ^*^P < .05, ^**^P < .01, and ^****^P < .0001.

To enhance our understanding of the essential role that microbiome signatures play in the response to VDZ in CD, we provided FMT to PGF mice suffering from colitis (Fig. 5A). First, the PGF mice were constructed. Utilizing 16S rRNA sequencing, our research revealed a significant reduction in the diversity of gut microbiota among PGF mice. This finding strongly suggests that a substantial number of bacterial populations have been diminished within these subjects (Fig. S6A and B). We then conducted sequencing of the baseline fecal samples obtained from clinical donors. The distribution of microbiota within the donor sample groups was consistent, but there were significant differences in the microbiota between groups (Fig. S5A and B), and the inter-group differences in the gut microbiota of clinical donors were consistent with the previous detection results of mouse fecal samples (Fig. S5C–H). Moreover, the results indicated that there were no significant differences between the clinical donors (baseline fecal samples) and recipient mice (post-FMT fecal samples). This observation suggests that the human gut microbiota was successfully transferred to the mice in both groups, indicating the efficacy of the microbiota transfer process (Fig. 5B and Fig. S7).

In the recipient mice, FMT from patients who were in remission resulted in several positive health outcomes. Specifically, these mice experienced an increase in body weight, which can be indicative of improved overall health and nutritional status (Fig. 5C). Additionally, there was a significant reduction in the DAI scores, suggesting that the severity of the disease was diminished in these animals (Fig. 5D). Furthermore, the pathological symptoms that were initially present showed considerable alleviation, enhancing the well-being of the recipient mice (Fig. 5E and F). In contrast, FMT derived from patients who were not in remission did not demonstrate any discernible effects on the treatment outcomes of the recipient mice. In the cohort receiving FMT that achieved remission, there was a significant increase in the expression levels of ZO-1 and occludin (Fig. 5G–I). This finding indicates a significant enhancement in the integrity and functionality of the intestinal barrier. The study also revealed considerable changes in the levels of serum inflammatory cytokines (Fig. 5J). Despite this, the indicators that were discussed earlier displayed only slight variations within the FMT-non-remission cohort. To summarize, the initial composition of the gut microbiota was characterized by the presence of dominant bacterial species recognized for their anti-inflammatory characteristics. As a result, this specific microbiota profile contributed to an improved response to therapeutic interventions.

### Baseline gut microbiota–derived LCA unveiled as a potential noninvasive predictor for VDZ response in CD

**Figure 6:**
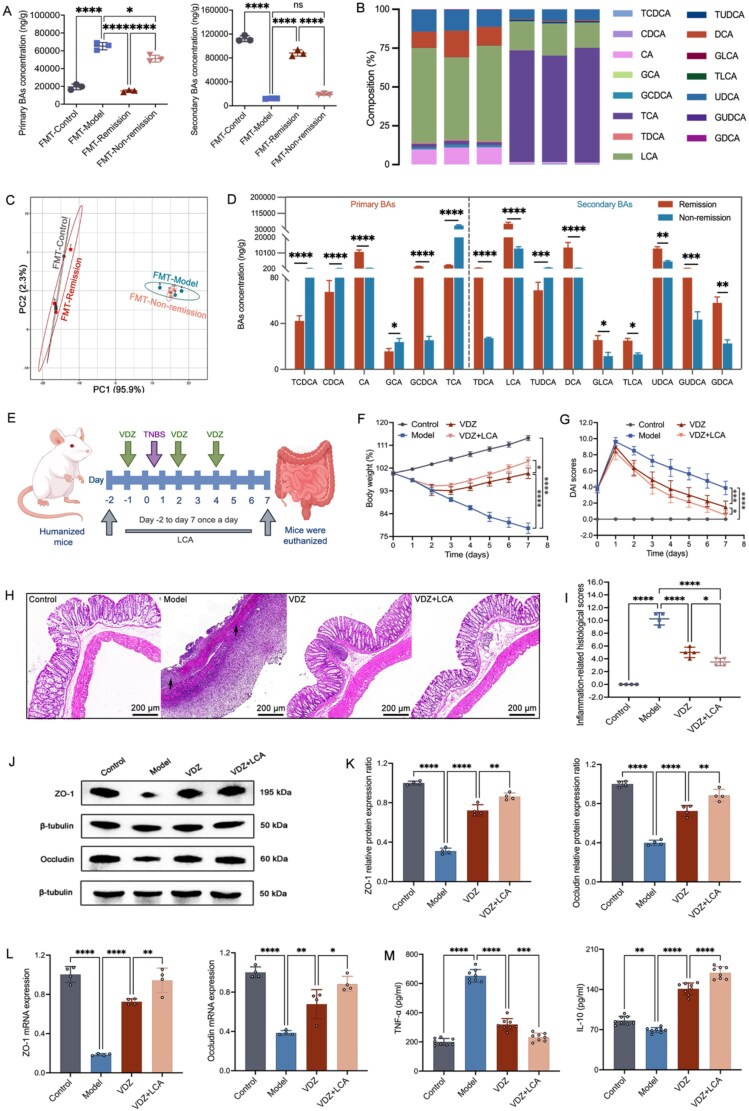
Gut microbial metabolite LCA associated with treatment outcomes in CD. (A) The concentration of primary and secondary BAs (*n* = 3); (B) composition of BAs (*n* = 3); (C) PCA score plot visually represents the key dimensions of variability in BA composition among the samples (*n* = 3); (D) comparative analysis of the relative diversity of significantly altered BAs (*n* = 3); (E) schematic diagram of TNBS-induced inflammation and VDZ and LCA intervention experiments. (F) Body weight change over time (*n* = 8); (G) DAI over time (*n* = 8); (H and I) illustrative images with a scale bar of 200 μm and associated scores of H&E (inflammatory cell infiltration indicated by black arrows, *n* = 4); (J and K) illustrative immunoblots along with the protein levels of *ZO-1* and *occludin* (*n* = 4); (L) tight junctions’ mRNA levels (*n* = 4); (M) serum levels of inflammatory cytokines (*n* = 8). Results are expressed as mean ± SD. The one-way analysis of variance and Student’s *t*-test were applied. ^ns^*P* > .05, ^*^P < .05, ^**^P < .01, ^***^P < .001, and ^****^P < .0001.

Given the crucial involvement of BAs in initiating intestinal inflammation [40], our investigation aimed to examine the potential impact of these BAs on treatment outcomes. In the course of this research, we assessed BA levels following FMT through targeted metabolomics analyses of fecal samples. The principal component analysis revealed a distinct clustering of BAs within each of the defined groups. There was a considerable distance separating the clusters corresponding to the remission group and the non-remission group (Fig. 6C). Compared with the non-remission mice, the remission mice had lower primary BAs and higher secondary BAs (Fig. 6A). Specifically, LCA constituted the major quantitative component of these secondary BAs’ signature associated with the remission phenotype (Fig. 6B and D), which is consistent with the previous serum untargeted metabolomics results. To further confirm the regulatory effect of LCA on VDZ treatment response in CD, we directly administered LCA to CD mice (Fig. 6E). Compared with the VDZ group, the VDZ + LCA group showed a significant increase in body weight and a significant decrease in DAI scores (Fig. 6F and G). Besides, the VDZ + LCA group showed recovery of intestinal barrier damage, and the level of intestinal inflammation significantly decreased (Fig. 6H–M). Consequently, our findings indicated that microbial diversity associated with LCA played a role in the remission of CD. The results presented here support the proposed mechanism by which LCA enhances the response to VDZ.

### LCA is mechanistically linked to the remission of CD by directly forming a pro-resolutive intestinal macrophage niche

**Figure 7:**
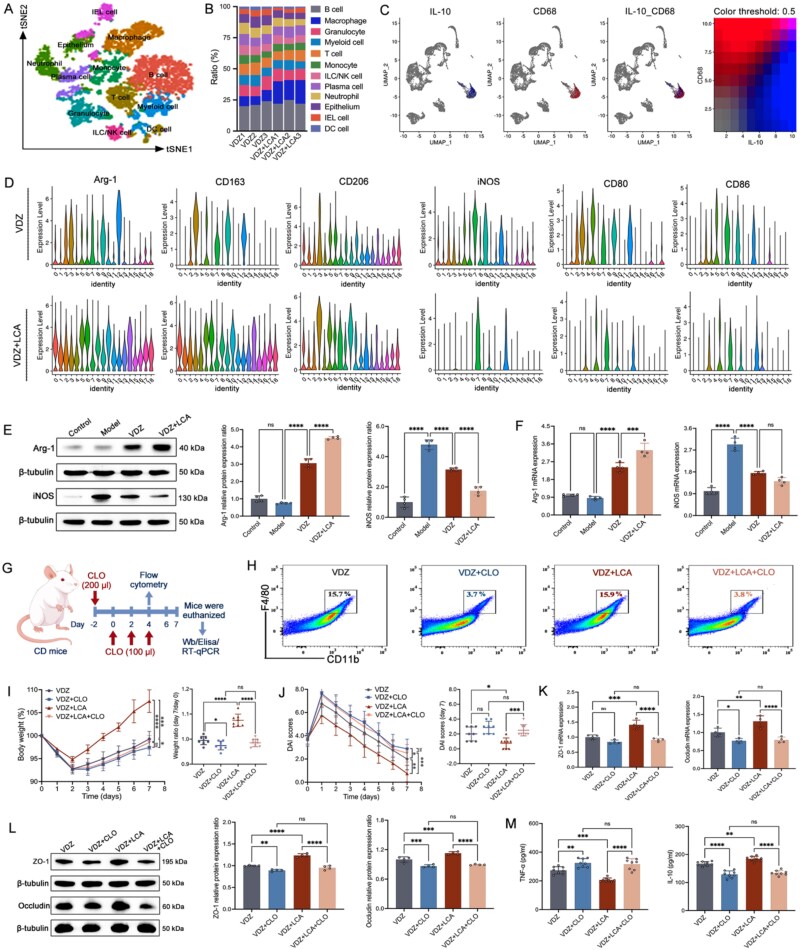
Intestinal macrophages are important mediators of LCA regulation of the VDZ treatment response in CD. (A) t-SNE visualization of single-cell transcriptomes (*n* = 3); (B) cell composition of different samples (*n* = 3); (C) predicted ligand–receptor interactions via CellChat analysis (*n* = 3); (D) violin plots depicting the marker expression associated with each macrophage cell cluster (*n* = 3); (E) immunoblots and quantitative results of macrophage polarization (*n* = 4); (F) the mRNA levels of macrophage polarization (*n* = 4); (G) schematic diagram of the CLO depletion experiment on macrophages in CD mice; (H) the clearance effect of macrophages was assessed using flow cytometry at day 4 (*n* = 4); (I) body weight change and day 7 relative weight (*n* = 8); (J) DAI over time and on day 7 (*n* = 8); (K) tight junctions mRNA levels (*n* = 4); (L) illustrative immunoblots along with the protein levels of *ZO-1* and *occludin* (*n* = 4); (M) serum levels of inflammatory cytokines (*n* = 8). Results are expressed as mean ± SD. A one-way analysis of variance was utilized. ^ns^*P* > .05, ^*^P < .05, ^**^P < .01, ^***^P < .001, and ^****^P < .0001.

To explore the specific mechanism of LCA promoting treatment response, we selected colon tissue with VDZ group and VDZ + LCA group for single-cell RNA sequencing. A total of 10 825 individual cells linked to fractures were sequenced, and after thorough filtration, 9957 of these were kept for further analysis. Twelve distinct cell clusters were identified using *t*-distributed stochastic neighbor embedding. These clusters include B cell, macrophage, granulocyte, myeloid cell, T cell, monocytes, ILC/NK cell, plasma cell, neutrophil, epithelium, IEL cell, and DC cell (Fig. 7A). Substantial variations were observed in the quantity of these 12 cell clusters across the two groups (Fig. 7B). Correlation analysis indicated that macrophages may be involved in treatment response (Fig. 7C). Macrophages are key mediators of the immune system and are found throughout the gastrointestinal tract. Increasing evidence supports that macrophages play a crucial role in the pathology of CD. Therefore, we conducted a subsequent subtype analysis of macrophages and carefully identified its surface markers. Both the dot plot and violin plot show the expression levels of macrophage markers in each cell cluster, and the consistency between these two results indicates that in the VDZ + LCA group, the surface markers of macrophage group *Arg-1*, *CD206*, and *CD163* were significantly increased, while *iNOS*, *CD86*, and *CD80* were significantly decreased (Fig. 7D and Fig. S8).

We then conducted *in vivo* experiments, and the results from WB and RT-qPCR indicated a significant enhancement in the levels of the M2 marker, but a marked decrease in the expression of the M1 marker was observed in the VDZ + LCA group (Fig. 7E and F). Furthermore, we used CLO to deplete macrophages in humanized mice to establish a CD model and intervened with VDZ and VDZ combined with LCA, respectively (Fig. 7G). FC analysis revealed a significant reduction in this percentage in both CLO-treated groups compared to their respective non-CLO controls (15.7% vs. 3.7%; 15.9% vs. 3.8%), directly confirming efficient local macrophage depletion (Fig. 7H). The results showed that in CD mice with intestinal macrophages depleted, even when VDZ was used in combination with LCA, the treatment response level showed no significant difference compared to LCA alone (Fig. 7I–M). This indicates that intestinal macrophages are an important mediator through which LCA regulates the therapeutic response to VDZ in CD, although the specific signaling pathway involved still needs to be investigated.

### LCA drives macrophage polarization to the M2 subtype via the synergistic TGR5/FXR

**Figure 8:**
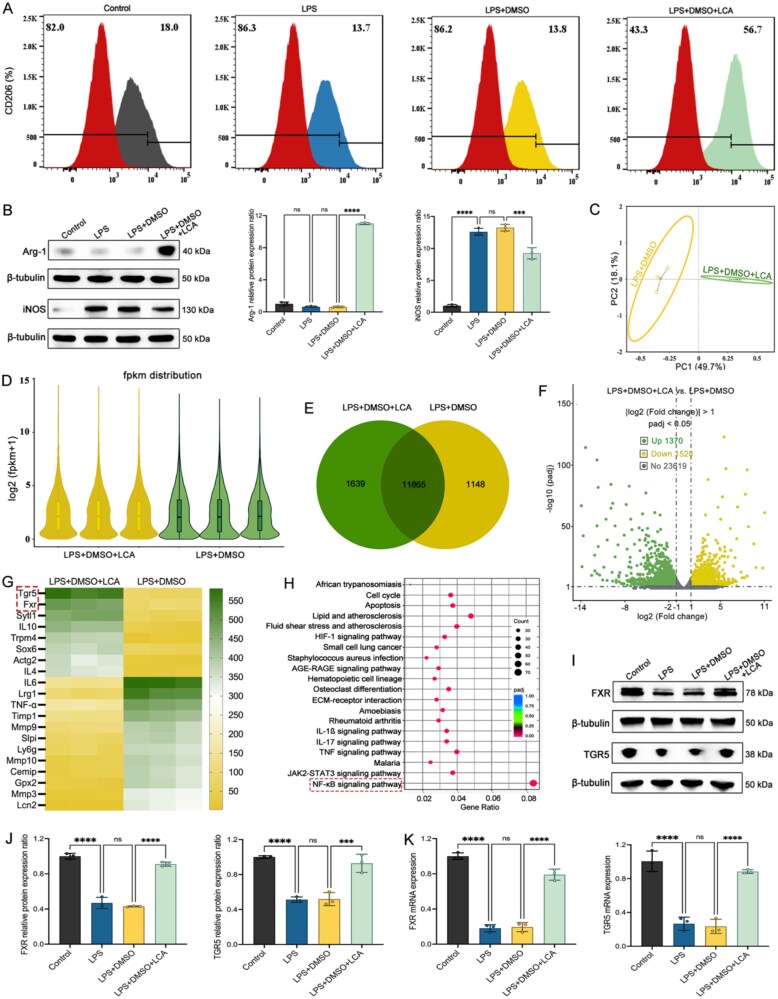
Gut microbiota–related metabolism LCA–*TGR5*/*FXR* axis impacts in response to VDZ in CD. (A) Flow cytometry results of CD206 in each group; (B) immunoblots and quantitative results of macrophage polarization; (C) PCA score plot effectively summarizes the variations in gene expression profiles among the different groups; (D) gene expression distribution plot visually represents how genes are variably expressed across different conditions; (E) Venn diagram delineates the overlap and unique gene sets between the different experimental groups; (F) volcano plot shows which genes are significantly upregulated or downregulated; (G) correlation heatmap in the transcriptome; (H) top 20 representative pathways analyzed by the KEGG pathway method; (I and J) immunoblots and *TGR5* and *FXR* protein levels; (K) the mRNA levels of *TGR5* and *FXR* (*n* = 3 in each group). Results are expressed as mean ± SD. A one-way analysis of variance was utilized. ^ns^*P* > .05, ^***^P < .001, and ^****^P < .0001.

To explore the mechanism by which LCA regulates macrophage subtype transformation and thereby affects the response level, we performed transcriptome sequencing after treating THP-1 cells with LCA. We first used an LPS-induced inflammation model, and in the LCA intervention group, M2 markers were significantly enhanced compared to the other groups (Fig. 8A and B). Subsequently, transcriptome sequencing results indicated good reproducibility within groups and significant differences between groups. The expression levels of all genes in each sample were reasonably distributed, which met the expected results (Fig. 8C and D). Additionally, there is a significant difference in gene expression between the two groups (Fig. 8E and F). The gene showing the greatest difference between the two groups was *TGR5*, and the gene showing the second greatest difference was *FXR*. Both *TGR5* and *FXR* showed upregulated expression levels in the LCA intervention group (Fig. 8G). Besides, the enrichment results indicate that the nuclear factor kappa-B (NF-κB) signaling pathway has the largest proportion and the most significant differences (Fig. 8H).

**Figure 9:**
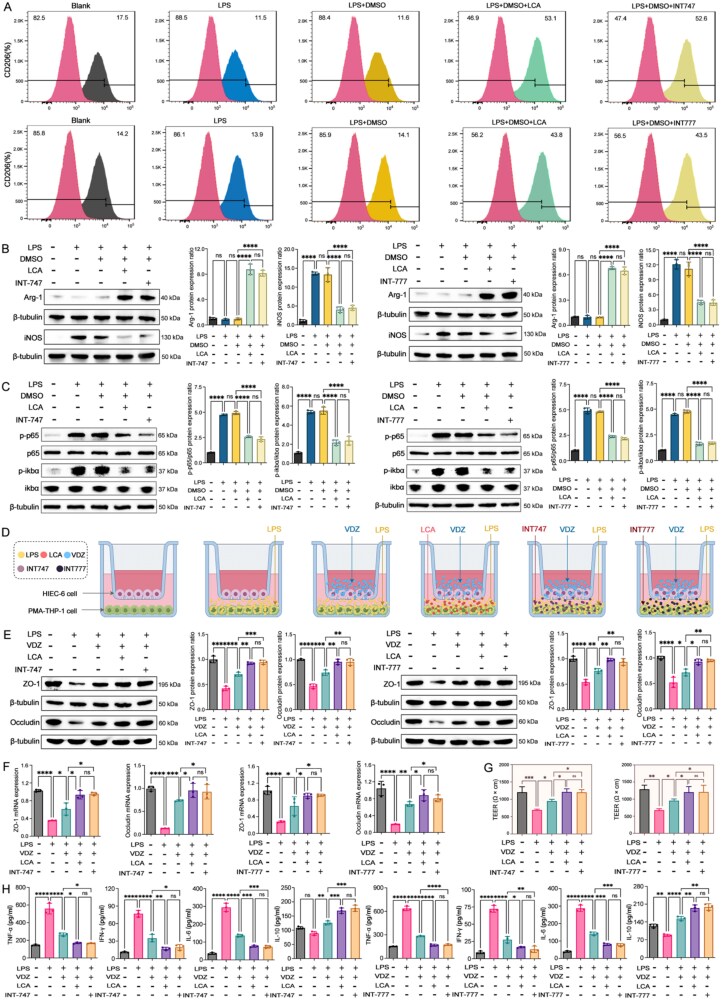
LCA enhances VDZ responsiveness in CD by shaping the pro-resolutive macrophage niche via the *TGR5*/*FXR*–NF-κB axis. (A) Flow cytometry results of CD206 in each group; (B) immunoblots and quantitative results of macrophage polarization; (C) immunoblots and quantitative results of NF-κB pathway; (D) schematic for the construction of the conditional medium cell co-culture system; (E) immunoblots and ZO-1 and occludin protein levels in co-culture system; (F) tight junctions’ mRNA levels in co-culture system; (G) the transendothelial resistance of the intestinal epithelium; (H) enzyme-linked immunosorbent assay of inflammatory cytokines (*n* = 3 in each group). Results are expressed as mean ± SD. A one-way analysis of variance was utilized. ^ns^*P* > .05, ^*^P < .05, ^**^P < .01, ^***^P < .001, and ^****^P < .0001.

Given that microbial BAs function as agonists for *FXR* and *TGR5* receptors and observing significant variations in LCA levels among different groups, we subsequently investigated if the activation of *FXR* and *TGR5* correlated with the response to VDZ in CD. There was a significant increase in the levels of *TGR5* and *FXR* expression in the LCA group (Fig. 8I–K). To investigate if LCA directly influences *FXR* and *TGR5*, THP-1 cells were treated with the INT747 (*FXR* agonist) and INT777 (*TGR5* agonist). The consistency of both flow cytometry and WB results indicates that the LCA’s regulatory ability on the macrophage M2 subtype transformation is consistent with that of the activator (Fig. 9A and B). So far, our study has confirmed the important role of the gut microbiota–BA axis in the therapeutic response of CD, but which signaling pathways are subsequently affected by changes in BA pool homeostasis and thereby influence the treatment response still require further in-depth investigation.

### LCA enhances VDZ responsiveness in CD by shaping the pro-resolutive macrophage niche via the *TGR5*/*FXR*–NF-κB axis

Activated NF-κB has been found in large amounts in macrophages and intestinal epithelial cells of patients with CD, suggesting that NF-κB has the ability to regulate the release of pro-inflammatory factors associated with the development of CD. Compared with the LPS-induced inflammation group, the NF-κB signaling pathway was significantly inhibited in both the INT747 and INT777 intervention groups, indicating that *FXR*/*TGR*5 exerts a negative feedback to regulate the NF-κB signaling pathway, which is consistent with the results of transcriptomic sequencing. Moreover, compared with the LPS-induced inflammation group, the inhibition of the NF-κB signaling pathway in the LCA intervention group was consistent with that of INT747 and INT777, indicating that LCA can affect the NF-κB signaling pathway by regulating *FXR*/*TGR5* (Fig. 9C).

To further verify whether LCA can influence the NF-κB signaling pathway through *FXR*/*TGR5* and thereby regulate the response level to VDZ treatment, we established a conditioned medium co-culture system with the specific construction process (Fig. 9D). Compared with the VDZ monotherapy group, the VDZ combined with the LCA intervention group showed a significant reduction in inflammation, marked repair of intestinal barrier damage, and treatment efficacy consistent with the activator, indicating that the gut microbiota metabolite LCA–*FXR*/*TGR5*–NF-κB cascade axis is a potential mechanism influencing the response of CD to VDZ (Fig. 9E–H). Therefore, LCA forms a microbiota-immune circuit, which reprograms macrophages into a pro-resolutive phenotype via the *TGR5/FXR*–NF-κB signaling network, thereby enhancing the responsiveness of CD to VDZ.

## Discussion

CD is a complex chronic transmural inflammatory bowel disease, clinically characterized by progressive intestinal stenosis, penetrating lesions, and irreversible fibrosis, with a high-risk recurrence tendency. In recent years, the rapid development of biological agents has significantly improved the efficacy of CD. Among them, VDZ is currently the only intestinal-selective biological agent. It can reduce the side effects of off-target effects outside the intestine and has a lower risk of infection and malignant tumors compared with other biological agents. At present, VDZ has been widely used in the clinical treatment of CD, but clinical response can only be achieved in about half of the individuals [41]. Similarly, our research found that only a portion of clinical CD patients could obtain therapeutic benefits after VDZ treatment. Although VDZ can significantly improve the current treatment situation of CD, there is still a phenomenon of no response to treatment, which seriously restricts its clinical application. Therefore, it is urgently necessary to explore the response mechanism of VDZ in treating CD, which is conducive to the construction of precise treatment to minimize the disease burden to the greatest extent.

In CD patients with different clinical activity stages, the degree of gut microbiota imbalance is closely related to the severity of the disease. Intestinal microbiologic disorders in CD patients may precede clinical symptoms, and 40% of CD patients have fecal flora disorders in their first-degree relatives [42]. Previous studies have shown that dynamic changes in the gut microbiota can reflect the therapeutic effect of CD patients. For example, in CD patients treated with anti–tumor necrosis factor-α, the accuracy of predicting efficacy using the baseline microbiome alone is better than that of traditional clinical markers [43]; in CD patients who experienced clinical remission after 6 weeks of induction treatment of ustekinumab, the alpha diversity of the intestinal microbiome is significantly higher than that of patients with active CD [44]. In addition, our study found that in the humanized mouse CD model, the baseline abundance of gut microbiota in the VDZ treatment remission group was significantly increased compared with the non-remission group, and the treatment response level of the recipient mice transplanted with clinically treated remission patients was significantly enhanced. The above shows that gut microbiota can affect the response level of the VDZ treatment of CD.

Under normal circumstances, the interaction between gut microbiota and BAs plays a crucial role in maintaining the homeostasis of the host. However, the interaction between gut microbiota and BAs under specific pathological conditions can have adverse effects on the biotransformation process in the host. This is also considered to be one of the key factors in the occurrence and development of CD. With the widespread application of gut microbiota sequencing technology and BA mass spectrometry, metabolomics research related to gut microbiota is gradually carried out [45]. A previous investigation found that in patients with CD, the ratio of *F. prausnitzii* to *E. coli* decreased, thereby weakening the deconjugation effect of gut microbiota, resulting in a significant increase in conjugated BAs in feces [40]. Previous research results also showed that the reduction of *Clostridium* sp. XIVa in CD patients would lead to a reduction in the ratio of deoxycholic acid and cholic acid in serum and feces [46]. Moreover, the decrease in the number of *Clostridium tenellae* was positively correlated with the lack of deoxycholic acid and LCA in feces [47]. In our study, the results of targeted BA metabolomics testing showed that in the remission group, higher microbiota enzyme activity could cause an increase in LCA and promote CD treatment response by reducing intestinal inflammation and improving intestinal mucosal barrier function.

The interaction between BAs and immune cells is critical to the intestine, and multiple existing evidence indicate that BAs play a key role in affecting mucosal immune cell dynamics [48]. The derivatives of LCA and deoxycholic acid play a crucial role as signaling molecules that critically affect the differentiation processes of TH17 and Treg cells. These processes are essential in the context of immune responses, particularly in the regulation of intestinal inflammation [49–51]. The collaboration of 5α/β-reductase and 3α/β-HSDH results in the formation of various derivatives of LCA and DCA, such as iso-, 3-oxo-LCA/DCA, allo-, 3-oxoallo-, and isoalloLCA. 3-oxo-LCA directly interacts with RORγt, preventing TH17 cell differentiation, whereas isoalloLCA boosts *FOXP3* expression by producing mitochondrial reactive oxygen species, which promote the development of resilient inflammatory Treg cells [49]. A subsequent investigation revealed that IsoalloLCA enhanced the interaction of *NR4A1* with the *FOXP3* region, leading to improved transcription of the *FOXP3* gene and consequently facilitating the differentiation of Treg cells [50]. Besides T lymphocytes, macrophages represent another crucial element of the immune system. They can exhibit a pro-inflammatory M1 phenotype triggered by the classical pathway or an anti-inflammatory M2 phenotype stimulated by the alternative pathway [52]. In our study, we found that the regulatory effect of LCA on CD treatment response is intimately linked to the promotion of intestinal macrophage differentiation toward an M2-resolutive phenotype.

**Figure 10:**
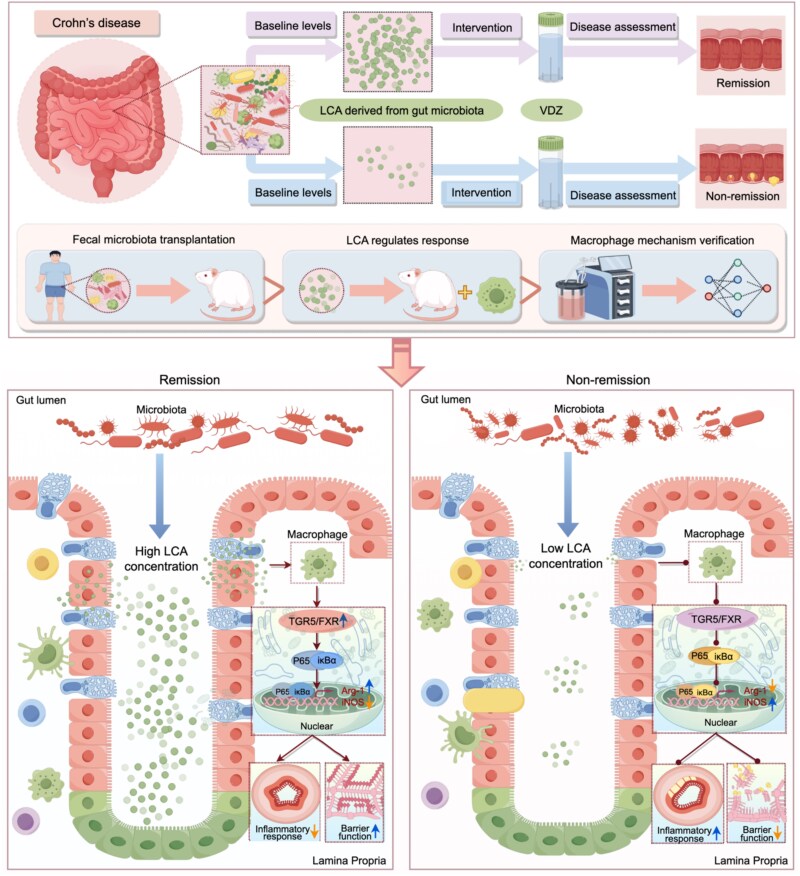
Illustration of the mechanism impacting response to VDZ in CD. Baseline gut microbiota of CD mice contains common microbes that modulate microbial secondary BA levels. The increased BA levels, especially LCA, led to further improving the response level. LCA can target intestinal macrophages and specifically activate *TGR5* and *FXR*. Furthermore, TGR5/FXR collaboratively regulates the inflammatory response and intestinal barrier function by inhibiting the NF-κB signaling pathway to reshape the intestinal mucosal immune microenvironment, ultimately improving the response level. Figure 10 is drawn using the online web page Figdraw.

BA receptors play a role in regulating immune cells in the intestine. Additionally, isoDCA, a derivative of DCA, enhances the induction of Foxp3 by impairing the immune-stimulating functions of DC cells, resulting in the expansion of Treg cells in the colon [51]. The engineered consortium that produces isoDCA was transplanted into sterile mice to promote the production of colon RORγ^+^ Treg cells in a CNS1-dependent manner [51]. Ablation of *FXR* in DC cells results in a similar transcription profile to isoDCA and enhances the production of peripheral Treg cells. This suggests a collaborative role between *FXR* and isoDCA in sustaining the anti-inflammatory characteristics of DC cells [51]. Bacteroides were introduced into germ-free mice, resulting in the induction of colon RORγ^+^ Treg cells through *VDR* and *FXR*. However, the genetic disruption of the BA deconjugating enzyme BSH within Bacteroides considerably hindered this induction [22]. The restoration of the intestinal BA pool enhanced the population of RORγ^+^ Tregs and increased the host’s vulnerability to inflammatory colitis when subjected to a low dietary regimen (limited nutrition), simultaneously diminishing the impact of dietary components on the Treg population [22]. These studies confirm the BA receptor–mediated regulatory effect of BAs on Treg cell populations.

The involvement of *FXR* in CD has been explored in both human subjects and mouse models. A newly developed fibroblast growth factor 19 (*FGF19*) analog has been shown to safeguard mice against intestinal inflammation while also altering the composition of the microbiota. This anti-inflammatory effect was entirely eliminated in mice lacking *FXR* [53]. However, it was also discovered that the conjugation of cholate, a BA, with specific amino acids such as phenylalanine and tyrosine—designated as Phe-chol and Tyr-chol—exhibits strong agonistic activity toward *FXR*. These conjugated forms were found to be significantly present in dysbiotic states, which are often observed in patients who are affected by CD. This suggests that although *FXR* and *FGF19* may offer protective effects, the presence of potent *FXR* agonists linked to certain amino acid conjugates could complicate the disease landscape in CD patients [54]. *TGR5* represents a significant BA receptor that plays a role in the development of colitis. By activating the proto-oncogene tyrosine-protein kinase/yes-related protein 1 regeneration pathway, LCA and DCA facilitate the regeneration of intestinal organoids and stimulating *TGR5* in intestinal stem cells (ISCs) [23]. Mice that have *TGR5* disrupted in their ISCs experience more severe colitis compared to the wild-type group. Furthermore, the stimulation of *TGR5* through the agonist INT-777 enhances the reconstitution of stem cells, indicating that TGR5 plays a crucial role in the regeneration of the intestinal epithelium [47]. Additionally, another investigation revealed that LCA suppressed the activation of the *NLRP3* inflammasome via the *TGR5*-*cAMP*-*PKA* pathway, leading to enhanced management of inflammation [55].

Our study revealed that the gut microbiota–LCA axis mediates the negative feedback of *FXR*/*TGR5* to regulate the NF-κB signaling pathway, thereby improving the therapeutic response level of CD to VDZ. NF-κB in an activated state has been found to be present in large quantities on macrophages and intestinal epithelial cells in CD patients, which means that NF-κB could regulate the release of pro-inflammatory factors associated with CD development. Cheon *et al*. found that plant steroids can reduce the inflammatory response in colitis mice. The main mechanism is that plant steroids can hinder the *IκK* kinase activity of intestinal epithelial cells, thereby blocking the activation of NF-κB signal transduction pathway, thereby exerting an anti-inflammatory effect [56]. In addition, researchers synthesized a special oligonucleotide that binds NF-κB and blocks the associated inflammatory transmitters. The use of this oligonucleotide in the intestine can reduce the severity of the disease, improve colon pathological changes, and reduce the levels of pro-inflammatory cytokines [57]. Briefly, this approach has shown significant effects in the field of CD therapy by interrupting the activation pathway of NF-κB to alleviate disease symptoms.

We systematically explore the response mechanism of CD to VDZ treatment. Baseline gut microbiota of CD mice contains common microbes that modulate microbial secondary BA levels. The increase in secondary BA levels, especially LCA, can target intestinal macrophages and specifically activate *TGR5* and *FXR*. *TGR5*/*FXR* further collaboratively regulates inflammatory response and intestinal barrier function by inhibiting the NF-κB signaling pathway to reshape the intestinal mucosal immune microenvironment, ultimately improving the response level (Fig. 10). Although our study delineates a microbiota-immune circuit influencing VDZ responsiveness, several limitations should be considered. First, the fecal microbiota transplantation experiments were derived from a limited number of clinical donors. While this focused design was instrumental in identifying LCA as a strong candidate metabolite, future studies with larger, diverse cohorts are necessary to validate its predictive value and generalizability across the heterogeneity of CD. Second, although we provide multifaceted evidence supporting the *TGR5*/*FXR*–NF-κB axis in macrophages, definitive *in vivo* causality using myeloid-specific knockout models would strengthen the mechanistic claim and represents a critical next step. LCA is also known to interact with other nuclear receptors, including *VDR*. Although our data point to a dominant role for *TGR5*/*FXR* signaling under our experimental conditions, the potential contribution of parallel or complementary pathways warrants future investigation. Third, the translational interpretation of our findings must consider the constraints of the humanized mouse model. The reconstituted human immune system is a simplification, and the acute TNBS-induced colitis does not fully mirror chronic human disease. Nevertheless, this model provided a unique and controlled platform to demonstrate that a gut microbial metabolite can directly engage human immune cells to influence therapeutic outcomes. The mechanistic pathway elucidated here establishes a framework for future validation in patient-derived cells and tissues.

## Conclusion

Taken together, gut microbial metabolite LCA enhances VDZ responsiveness in CD by shaping the pro-resolutive macrophage niche via the *TGR5*/*FXR*–NF-κB axis. At present, establishing a comprehensive response prediction model remains a challenging task. Integrating multiomic data for molecular phenotype analysis can help us better understand the regulatory mechanisms of CD treatment response, so as to more accurately formulate the most suitable treatment plan for clinical patients. As the functional characteristics of the gut microbiota change, the composition of BA pools in CD patients will also change. In-depth discussion of the gut microbiota and BA changes in CD patients and their related signaling pathways in CD treatment response will provide more new perspectives for the clinical treatment of CD and help develop personalized disease management strategies. Furthermore, although this study was explicitly designed to address VDZ responsiveness in CD, the elucidated LCA–macrophage–*TGR5*/*FXR* axis comprises conserved immunometabolic pathways. Future investigations are warranted to examine whether analogous microbial or metabolomic determinants exist and modulate VDZ efficacy in the distinct clinical and immunological context of ulcerative colitis.

## Supplementary Material

Supplymentary_Material_wrag028

## Data Availability

These mNGS, 16S rRNA, transcriptome, and single-cell RNA sequencing data have been submitted to NCBI (www.ncbi.nlm.nih.gov) and can be accessed using the following numbers: PRJNA1332501, PRJNA1333210, PRJNA1333309, PRJNA1333332, and PRJNA1418134.
