# Supplementary material for: Gut microbial production of lithocholic acid reprograms pro-resolutive macrophages to enhance vedolizumab responsiveness via the TGR5/FXR–NF-κB axis
Source: ISME J. 2026 Feb 16;20(1):wrag028. doi: 10.1093/ismejo/wrag028 (PMC12998432; doi:10.1093/ismejo/wrag028)
Supplement: Supplymentary_Material_wrag028 [file supplymentary_material_wrag028.docx]

**Materials and methods**

**1. 2,4,6-trinitrobenzene sulfonic acid (TNBS)-induced colitis**

TNBS-induced colitis shares similar immunological and histopathological properties and is therefore widely used to study different aspects of CD ^[1, 2]^. TNBS was sourced from Sigma-Aldrich (Cat #40-4700; MO, USA). After a 7-day adaptation period, mice were randomly assigned. To induce colitis, administer TNBS as described above ^[2]^. In summary, mouse skin was pre-sensitized by applying 150 μl of a 1% (wt/vol) TNBS-olive oil-acetone solution over a period of 7 days. Following a 24-hour fasting period, the mice received a colonic injection of 100 μl of 2.5% (wt/vol) TNBS dissolved in 50% ethanol. Healthy mice received the equal amount of phosphate-buffered saline (PBS). PBS was sourced from Servicebio (Cat #G4202; Wuhan, China).

**2.** **Establishment of humanized gut microbiota**

Fecal microbiota transplantation (FMT) corresponds to the original description, but with some minor changes ^[3]^. All donor patients diagnosed with CD were included to ensure a more thorough and precise evaluation of treatment response. The criteria for inclusion were applicable to all CD patients initiating VDZ within the framework of their standard clinical therapy. Patients received an intravenous dose of 300 mg of VDZ at weeks 0, 2, and 6, with subsequent doses given every 8 weeks thereafter. Patients who have undergone ileostomy procedures or have J-pouches were deliberately excluded from the research due to the challenges associated with accurately assessing disease activity in these particular populations. The result achieved at week 14 was deep remission without the use of corticosteroids (SFDR). This SFDR was characterized by a combination of factors: a C-reactive protein concentration at or below 5 mg/L, a fecal calprotectin measurement of 150 μg/mg or less, a Harvey Bradshaw index score under 5, and an Inflammatory Bowel Disease Questionnaire score exceeding 170 ^[4]^. The study comprised a total of 30 individuals with CD. Among these participants, 10 did not receive treatment with VDZ, while another 10 patients were in remission after undergoing VDZ therapy. Additionally, there were 10 patients who continued to experience symptoms and remained in a non-remission state despite having received the treatment. Moreover, the research involved 10 healthy participants as well. Detailed characteristics of the donors can be found in Table S1. Donors were matched in terms of age, gender and medication history. All individuals provided written informed consent. The research protocol received approval from the Ethical Committee of the First Affiliated Hospital of Zhengzhou University (Approval Number: 2025-KY-0898).

Fresh fecal samples from the above donors were immediately processed in an anaerobic chamber (Coy Laboratory Products) within 15 minutes of collection to minimize oxygen exposure. An equal mass of feces (500 mg) from each donor was homogenized (1:5, w/v) in sterile, pre‑reduced phosphate‑buffered saline supplemented with 15% (v/v) glycerol as a cryoprotectant. The resulting suspension was passed through a sterile 100 μm cell strainer, aliquoted into cryovials, flash frozen in liquid nitrogen, and stored at -80°C. For transplantation, a precise volume of 200 μl of the prepared fecal suspension was administered to each recipient mouse. This protocol is optimized to maintain microbial viability and structural integrity ^[3]^. The fecal suspensions of all donors were randomly assigned to the cages of recipient mice by computer-generated sequences. All personnel involved in mouse rearing, FMT administration, phenotypic assessment, and sample collection/processing were blinded in donor outcome groups. Blind code is independently saved and maintained until the final data analysis. For a given donor group, the suspensions from all 10 donors were pooled together in equal volumes to create a group-specific FMT inoculum. To prevent cross-contamination of donor-specific microbiota, this pooled inoculum was then administered to recipient mice that were co-housed in a single cage.

**3. Evaluation of the general situation**

Weight measurements for the mice were collected each day, and any indications of hidden or significant rectal bleeding, along with the nature of their stool consistency, were documented. The DAI is computed on a daily basis for every laboratory animal by taking the total of these scores and dividing it by three, as outlined earlier ^[5]^. Following the euthanasia of the mice, intestinal dissection was conducted to evaluate intestinal damage, and the colonic mucosa damage index (CMDI) score was determined based on a standardized protocol ^[6]^. Ultimately, a segment of the distal colon measuring 1 cm in length was collected for histological evaluation, immunohistochemical analysis, and immunofluorescence staining. The remaining colon was immediately frozen with liquid nitrogen to facilitate molecular analysis. The serum was frozen for additional examination of inflammatory cytokines.

**4. Histological assessment**

After sampling the mice, the colon tissues were rinsed clean in pre-cooled PBS, dried with clean filter paper, and then fixed in 4% paraformaldehyde for more than 24 hours. Subsequently, they were subjected to gradient dehydration and then embedded in paraffin. Finally, the wax blocks were cut to 5 μm and stained with H&E. Following this, a standard procedure was used to evaluate the histological scores related to inflammation ^[7]^. The total histological damage score was calculated as the sum of subscores from five parameters: goblet cell depletion (presence = 1, absence = 0), crypt abscesses (presence = 1, absence = 0), mucosal architecture destruction (normal = 0, mild = 1, moderate = 2, extensive = 3), muscle thickening (normal = 0, mild = 1, moderate = 2, extensive = 3), and cellular infiltration (normal = 0, mild = 1, moderate = 2, transmural = 3).

**5. Immunofluorescence**

The samples were incubated overnight at 4℃ with primary antibodies such as ZO-1 (Cat #61-7300) and occludin (Cat #40-4700). Subsequent to washing with PBS, incubate with secondary antibodies (Cat #A27034 and Cat #A11012). All the antibodies were obtained from Invitrogen (CA, USA). Following counterstaining with DAPI, the samples were analyzed using laser scanning confocal microscop (Leica, Wetzlar, Germany).

**6. Immunohistochemistry**

The experimental procedure involved the initial step of placing the samples in an incubator alongside the primary antibody for MPO (Cat #PA5-16672). Following this incubation period, the slides were subjected to two wash cycles using phosphate-buffered saline (PBS) to remove excess antibody. Subsequently, the samples were treated with the secondary antibody (Cat #A24531). Ultimately, a counterstaining with hematoxylin was performed, followed by dehydration through a series of increasing alcohol concentrations, and the samples were subsequently covered with slide covers. Invitrogen (CA, USA) supplied both antibodies. Cell counting was carried out utilizing the ImageJ-Pro Plus (version 6.0).

**7. Measurement of inflammation cytokines**

The quantitative analysis of serum concentrations for TNF-α, IFN-γ, IL-6, and IL-10 was conducted utilizing enzyme-linked immunosorbent assay kits provided by R&D Systems (MN, USA). Absorbance measurements were taken at a wavelength of 450 nanometers to ascertain the cytokine levels in the samples. This analysis employed a microplate reader manufactured by Molecular Devices (Shanghai, China). Subsequently, the absorbance values were evaluated against a standard curve, which served as a benchmark for converting optical density readings into the actual concentrations of the analytes present in the serum samples.

**8. Real-time quantitative polymerase chain reaction (RT-qPCR)**

Colon tissue RNA was effectively isolated utilizing TRIzol reagent in accordance with the manufacturer’s guidelines, and its concentration was measured at 260 nm via spectrophotometry. Subsequently, complementary DNA was synthesized through a reverse transcription process utilizing the PrimeScript RT reagent (Cat #RR047A). Following the synthesis of complementary DNA, RT-qPCR was conducted using the Tli RNase H Plus reagent (Cat #RR820A). All reagents and primers were sourced from Takara Biotechnology (Shiga, Japan). The sequences of the primers used in this research are presented in Table S2. The experiments were carried out on the ProFlex PCR system, manufactured by Thermo Fisher Scientific (MA, USA).

**9. Western blot**

The extraction and quantification of proteins from colon tissue were carried out utilizing reagents from Thermo Fisher Scientific (Cat #89900, Cat #78430, and Cat #23227; MA, USA). The protein sample is separated by molecular weight through polyacrylamide electrophoresis, then transferred to a PVDF membrane, and then the target protein is specifically detected through the primary antibody/secondary antibody complex. The samples were incubated overnight at 4℃ with the following primary antibodies: ZO-1 (Cat #ab307799), occludin (Cat #ab216327), Arg-1 (Cat #ab124917), iNOS (Cat #ab178945), p65 (Cat #ab32537), p-p65 (Cat #ab76302), ikbα (Cat #ab32518), p-ikbα (Cat #ab133462), and β-tubulin (Cat #ab179513). Following the washing of the membranes, they were then incubated with the secondary antibody (Cat #ab6721) at room temperature. The antibodies were obtained from Abcam (Cambridge, UK). The SuperSignal West Pico PLUS chemiluminescent fluid (Thermo Fisher Scientific) was employed to visualize the membranes.

**10. Metagenomic high-throughput sequencing and data analysis**

The MolPure Stool DNA Kit (Cat #18820ES70; Yesen, Shanghai, China) is a commonly utilized and dependable tool for extracting genomic DNA. The NovaSeq PE250 (Illumina) was used as the sequencing tool, and the obtained sequencing data were applied to the subsequent bioinformatics analysis. Using the SOAPaligner software, the clean data were compared in detail with the existing microbial sequences in the NCBI database. Then, using the successfully aligned read data, abundance statistics were conducted for different classification levels. The MetaGeneMark was used for the predictive analysis of genes, and then the obtained.gff files were converted into.fNA gene sequence files. Gene sequences exceeding 100 bp were screened out from them and translated into the corresponding amino acid sequences. Principal component analysis was adopted when conducting diversity analysis, while multivariate statistical analysis tools Metastat and LEfSe were used for species with differences between groups ^[8]^.

**11.** **Non-targeted metabolomics and data analysis**

The analysis of metabolites was performed through serum metabolomic profiling on the Broad Metabolomics platform, utilizing liquid chromatography-mass spectrometry methods ^[9]^. Take 100 μl of the serum sample and add it to a centrifuge tube containing 300 μl of methanol. Shake thoroughly for 2 minutes, then let it stand for 10 minutes. Centrifuge at 12000 g for another 10 minutes. Transfer the supernatant to the injection bottle and then conduct the analysis. Firstly, PLS-DA analysis was conducted to screen out the metabolites with a VIP value greater than 1.5. The results showed that the metabolites with a P value less than 0.05 might be potential response markers. The correlation heatmap only showed the correlations between significantly altered metabolites. Subsequently, these metabolites were retrieved using the KEGG database, and relevant matches were conducted for the secondary spectra of the predicted metabolites.

**10. Correlation analysis between mNGS and non-targeted metabolomics**

The connection between metabolites and gut microbiota was assessed utilizing earlier techniques ^[10]^. The species abundance of the core intestinal microbiota at the genus level was regarded as an independent dataset, while the physicochemical index values of metabolites and the significant difference values were regarded as another independent dataset. The Pearson correlation coefficient was calculated using R software, and the Cytoscape software was used to visually analyze the interrelationship between the dominant genera and the differential metabolites. The enriched enzymes and their functions in the KEGG database were comprehensively summarized and comparatively analyzed, and a comprehensive response-related prediction model was established by applying binary Logistic regression analysis.

**12. 16S rRNA sequencing and data analysis**

The amplification universal primers were targeted to the v3-v4 region ^[11]^. Subsequently, the PCR products and AMPureXP magnetic beads were mixed in a 1:1 ratio, and then the screening operation was carried out. After mixing the samples, 1.8% agarose gel was used to recover the products, thereby achieving the complete construction of the library, and the NovaSeq PE250 (Illumina) sequencing platform was used to perform the double-ended sequencing operation. Finally, the phylogeny of dominant OTUs was analyzed using PyNAST software. The diversity was studied with the α-index analysis tool Mothur. Sample ranking analysis and cluster analysis were adopted to demonstrate β-diversity and explore the changes in species composition.

**13. Targeted BA metabolomics and data analysis**

Measurement was carried out using LC-MS technology. Initially, measure out 30 mg of the fecal sample, and subsequently incorporate 200 μl of pre-chilled ultrapure water for the homogenization process. Following this, introduce 1000 μl of pre-chilled methanol along with 10 μl of the internal standard. After thoroughly vortexing the mixture, incubate it at -20°C for 20 minutes to facilitate protein precipitation. Subsequently, centrifuge the mixture at 14000 g for 15 minutes in a refrigerated setting of 4°C, then carefully discard the upper liquid layer for vacuum drying. During the mass spectrometry analysis, a volume ratio of 1:1 of methanol to water was utilized for reconstitution. Again, under a 4°C environment, centrifugation at 14000 g was performed for another 15 minutes, and the resultant upper liquid layer was collected for sample analysis. The Multiquant software was employed to determine the chromatographic peak areas and their corresponding retention times. To evaluate the stability and reproducibility of the data, all samples were combined into an equal number of QC samples for thorough analysis.

**14. Single-cell RNA sequencing and data analysis**

Libraries for single-cell RNA sequencing were created utilizing a microfluidics-based method on the Chromium Single-Cell Controller (10X Genomics), following the guidelines provided by the manufacturers and employing the Chromium Single Cell 3’ Reagent Kit v3.1. Sequencing of the libraries was conducted using an NovaSeq 6000 (Illumina) machine. The Fastq files underwent processing via Cell Ranger (version 4.0.0), employing the default settings. Cell clusters were established using a marker-based manual annotation approach that had been previously applied to each dataset. Following this initial step, the Seurat object underwent conversion into the Scanpy format (version 1.6.0) with Seurat Disk (version 0.0.0.9019). Subsequently, all further analyses were conducted within a Python environment (version 3.6.10). The CellRank package (version 1.2.0) was utilized to merge RNA velocity data with transcriptomic similarity information into a unified kernel for the calculation. The estimator for generalized Perron cluster analysis was utilized to recognize macrostates. By examining the coarse-grained transition matrix, terminal states were deduced and subsequently applied to calculate absorption probabilities.

**15. Flow cytometry**

Colon tissue was collected to prepare a single-cell suspension, which was aliquot into flow cytometry tubes, with 100 μl per tube. Fluorescently labeled antibody was added, mixed well, and then placed at 4°C. After incubating in the dark for 30 minutes, 2 ml of PBS was added for washing. Gently pipette and mix the sample with a 1 ml micropipette tip, then centrifuge at 4℃, 1500 g for 5 minutes and discard the supernatant. Then, wash with 2 ml of PBS, centrifuge at 4°C, 1500 g for 5 minutes, and discard the supernatant. After fixation with 500 μl of 4% paraformaldehyde, the cell precipitate was sent into a flow cytometer for detection within 24 hours. The F4/80 (Cat #11-4801-85), CD11b (Cat #MA5-28274), and CD206 (Cat #17-2061-82) was identified. All the above three antibodies were purchased from Invitrogen. The analysis of these data were carried out with FlowJo v10.8.1 software.

**16. Cell culture and drug intervention**

The American Type Culture Collection (MD, USA) supplied the human THP-1 cell. The cells were maintained at 37°C in a 5% CO2 atmosphere using RPMI-1640 medium supplemented with 10% fetal bovine serum. When the cells achieved an approximate 80% confluence, they were passaged, and the media were refreshed every 2 days. THP-1 cells were usually induced to differentiate into macrophages with PMA (50 ng/mL；Cat #S1819, Beyotime Biotechnology), and then cultured with LPS (100 ng/mL; Cat #Y269298, Beyotime Biotechnology) for 48 hours while PMA was still present. For the administration of medications, cells were seeded at a density of 8×10^5^ cells per well and were treated with LCA (10 μM), INT777 (10 μM; Cat # HY-15677, MedChemExpress, NJ, USA), and INT747 (10 μM; Cat # HY-12222, MedChemExpress) for a duration of 12 hours. Cells were then extracted to obtain the RNA and proteins after being rinsed.

**17. Construction of co-culture system with conditioned medium**

Human HIEC-6 cell was provided by the American Type Culture Collection，and it was cultivated using a dedicated medium (DMEM high glucose + 10% FBS + 1% P/S). THP-1 cells and HIEC-6 cells were respectively inoculated in 6-well plates and cultured for 48 hours. Various types of THP-1 cells were stimulated with LPS for 48 hours, and 10 μM of LCA, INT777, and INT747 were respectively intervened for 12 hours. The supernatant of the culture medium was collected and centrifuged at 12000 g for 10 minutes to remove cell debris. Finally, the collected supernatant of the culture medium was used to stimulate HIEC-6 cells for 12 hours. Cells were then extracted after being rinsed.

**18. Transepithelial (TEER) resistance test**

Place the electrode in HBSS preheated to 37°C and equilibrate for 20 minutes. Remove the culture medium, add preheated HBSS, measure the transmembrane resistance value, and calculate the TEER value (TEER= measured resistance value - blank value). The TEER values were measured using Millipore Millicell ERS-3.0 to evaluate the health status and barrier function of the cellular layer. This experiment was tested using three independent biological samples.

**Reference**

[1] Kiesler P, Fuss IJ, Strober W. Experimental models of inflammatory bowel diseases. Cell Mol Gastroenterol Hepatol 2015;1(2):154-70. https://doi.org/10.1016/j.jcmgh.2015.01.006.

[2] Wirtz S, Popp V, Kindermann M, et al. Chemically induced mouse models of acute and chronic intestinal inflammation. Nat Protoc 2017;12(7):1295-309. https://doi.org/ 10.1038/nprot.2017.044.

[3] Yang M, Zheng XQ, Fan JJ, et al. Antibiotic-induced gut microbiota dysbiosis modulates host transcriptome and m6A epitranscriptome via bile acid metabolism. Adv Sci 2024;11(28):e2307981. http://doi.org/10.10

02/advs.202307981.

[4] Yarur AJ, Bruss A, Moosreiner A, et al. Higher intra-abdominal visceral adipose tissue mass is associated with lower rates of clinical and endoscopic remission in patients with inflammatory bowel diseases initiating biologic therapy: results of the constellation study. Gastroenterology 2023;165(4):963-75. https://doi.org/10.1053/j.gastro.20 23.06.036.

[5] Yousefi-Ahmadipour A, Rashidian A, Mirzaei MR, et al. Combination therapy of mesenchymal stromal cells and sulfasalazine attenuates trinitrobenzene sulfonic acid induced colitis in the rat: the S1P pathway. J Cell Physiol 2019;234(7):11078-91. https://doi.org/10.1002/jcp.27944.

[6] Millar AD, Rampton DS, Chander CL, et al. Evaluating the antioxidant potential of new treatments for inflammatory bowel disease using a rat model of colitis. Gut 1996;39(3):407-15. https://doi.org/10.113

6/gut.39.3.407.

[7] Chen G, Ran X, Li B, et al. Sodium butyrate inhibits inflammation and maintains epithelium barrier integrity in a TNBS-induced inflammatory bowel disease mice model. EBioMedicine 2018;30:317-25. <https://doi.org/10.1016/j.ebiom.2018.03>.030.

[8] Segata N, Izard J, Waldron L, et al. Metagenomic biomarker discovery and explanation. Genome Biol 2011;12(6):R60. https://doi.org/10.1186/gb-2011-12-6-r60.

[9] Franzosa EA, Sirota-Madi A, Avila-Pacheco J, et al. Gut microbiome structure and metabolic activity in inflammatory bowel disease. Nat Microbiol 2019;4(2):293-305. https://doi.org/10.1038/s41564-018-0306-4.

[10] Want EJ, Wilson ID, Gika H, et al. Global metabolic profiling procedures for urine using UPLC-MS. Nat Protoc 2010;5(6):1005-18. https://doi.org/10.1038/nprot.2010.50.

[11] [Elliott](https://pubmed.ncbi.nlm.nih.gov/?term=Elliott+PR&cauthor_id=6103229) PR, [Lennard-Jones](https://pubmed.ncbi.nlm.nih.gov/?term=Lennard-Jones+JE&cauthor_id=6103229) JE, [Hathway](https://pubmed.ncbi.nlm.nih.gov/?term=Hathway+N&cauthor_id=6103229) N. A simple index of Crohn’s-disease activity. Lancet 1980;1(8173):876. https://doi.org/10.101 6/s0140-6736(80)91372-0.

**Legends for Supplementary Figures**

**Figure S1.** **Flow cytometry verification of the construction results of humanized mice.** (A) Overall cell population detection map; (B) Single-cell population detection map; (C) Assume a two-dimensional scatter plot of the gate; (D) hCD45/(hCD45+mCD45) scatter plot. (n = 40 in each group).

**Figure S2. Flow cytometry verification of the of α4β7 expression on human immune cells.** (A) Control group; (B) Model group; (C) Remission group; (D) Non-remission group.

**Figure S3. Composition of gut microbiota in each group at baseline.** (A) Phylum level; (B) Class level; (C) Order level; (D) Family level; (E) Genus level; (F) Species level. (n = 6 in each group).

**Figure S4. Levels of bile acids and related enzymes in each group at baseline.** (A) Bile acid levels; (B) EC1.1.1.391 level; (C) EC1.1.1.52 level; (D) EC 3.1.2.26 level. (n = 6 in each group). Results are expressed as mean ± SD. A one-way analysis of variance was utilized. ^ns^*P* > 0.05, **P* < 0.05, ****P* < 0.001, and *****P* < 0.0001.

**Figure S5.** **Donor microbiota variability within groups.** (A) Principal coordinates analysis based on Bray-Curtis dissimilarity; (B) Differential abundance analysis; (A) The composition of gut microbiota at Phylum level; (B) The composition of gut microbiota at Class level; (C) The composition of gut microbiota at Order level; (D) The composition of gut microbiota at Family level; (E) The composition of gut microbiota at Genus level; (F) The composition of gut microbiota at Species level. (n = 3 in each group).

**Figure S6.** **Depletion of gut microbiota in recipient mice.** (A) PCA score plot are useful for visualizing alterations in microbial communities across various samples; (B) The α diversity is quantified by Chao 1, Shannon and Simpson indices to measure the richness and uniformity of species within microbial communities. (n = 3 in each group). Results are expressed as mean ± SD. The student’s t-test were applied. *****P* < 0.0001.

**Figure S7.** **Community α-diversity analysis of gut microbiota in recipient mice following FMT.** α-diversity indices of the gut microbiota within the same group of mice before and after receiving microbiota from human donors. Each panel displays the Chao1 index, Shannon index, and Simpson index. (A) Control group; (B) Model group; (C) Remission group; (D) Non-remission group. (n = 3 in each group). Results are expressed as mean ± SD. The student’s t-test were applied. ^ns^*P* > 0.05.

**Figure S8. Dot plot visualization of marker gene expression for macrophage subclusters identified by single-cell RNA sequencing.** (A) Dot plot showing the expression of canonical M2 macrophage marker genes across the five macrophage subclusters; (B) Dot plot showing the expression of canonical M1 macrophage marker genes across the same subclusters. (n = 3 in each group).

**Supplementary Figures**

**Figure S1**

**
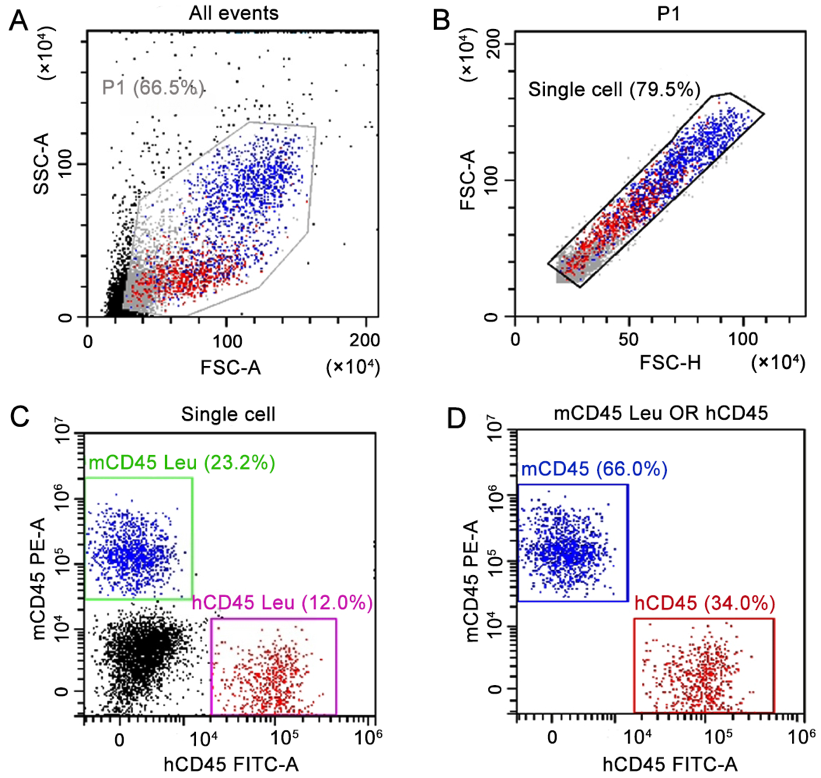
**

**Figure S2**

**
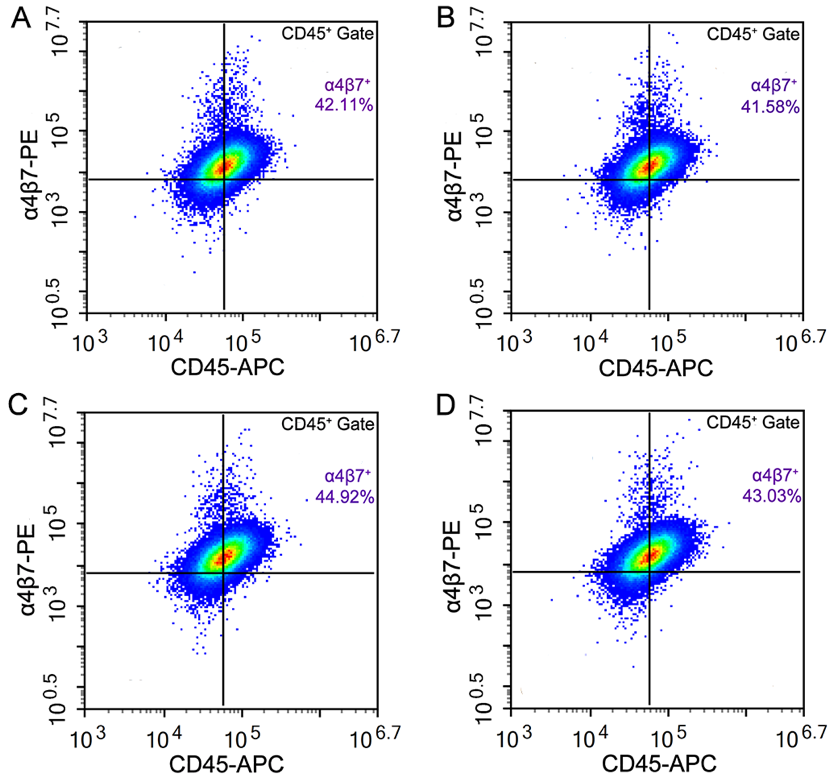
**

**Figure S3**


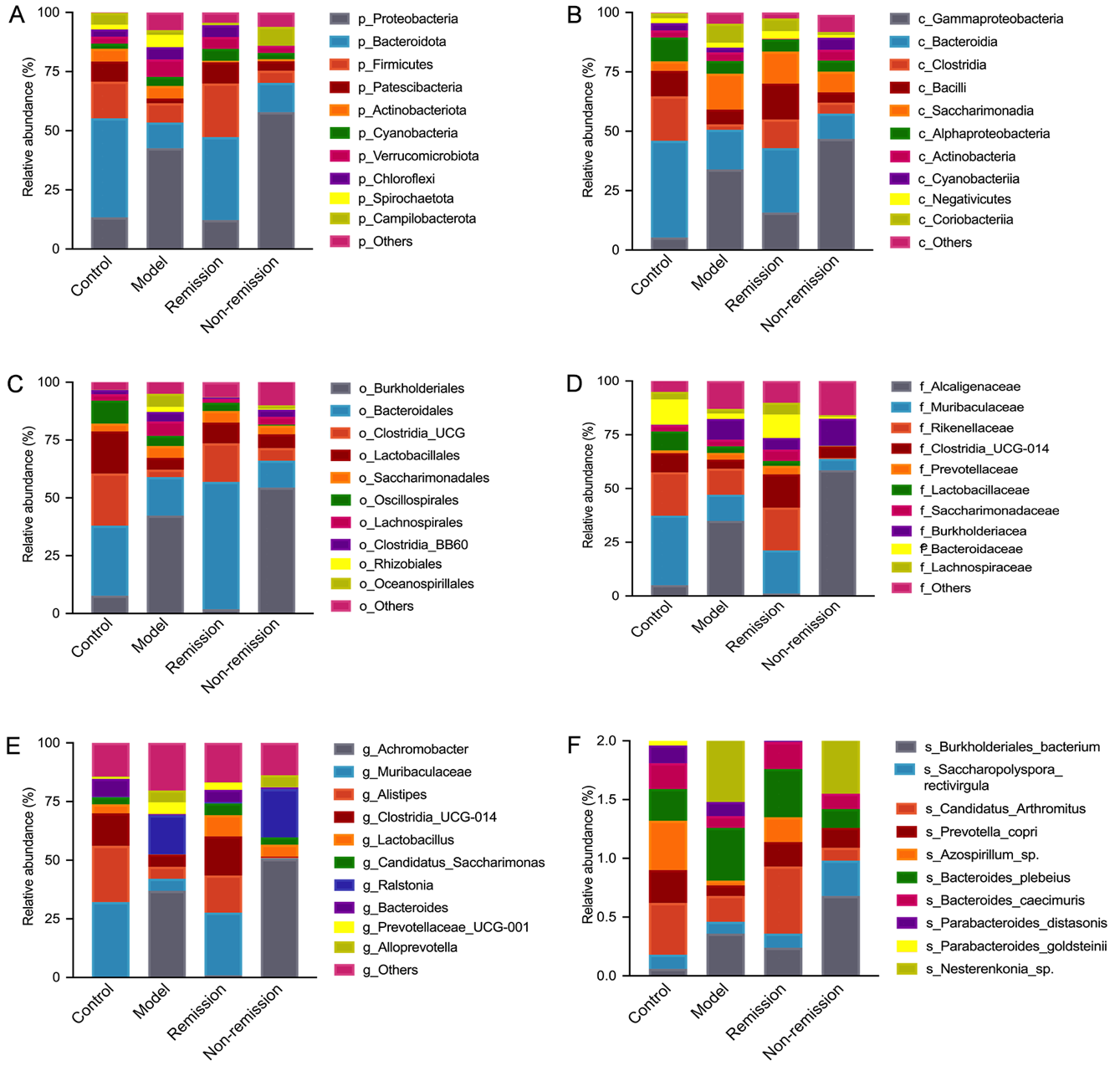


**Figure S4**


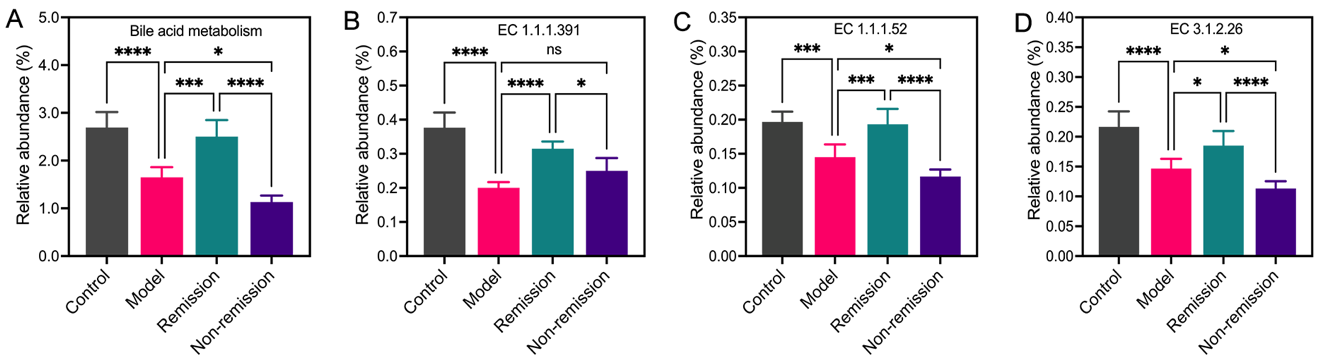


**Figure S5**


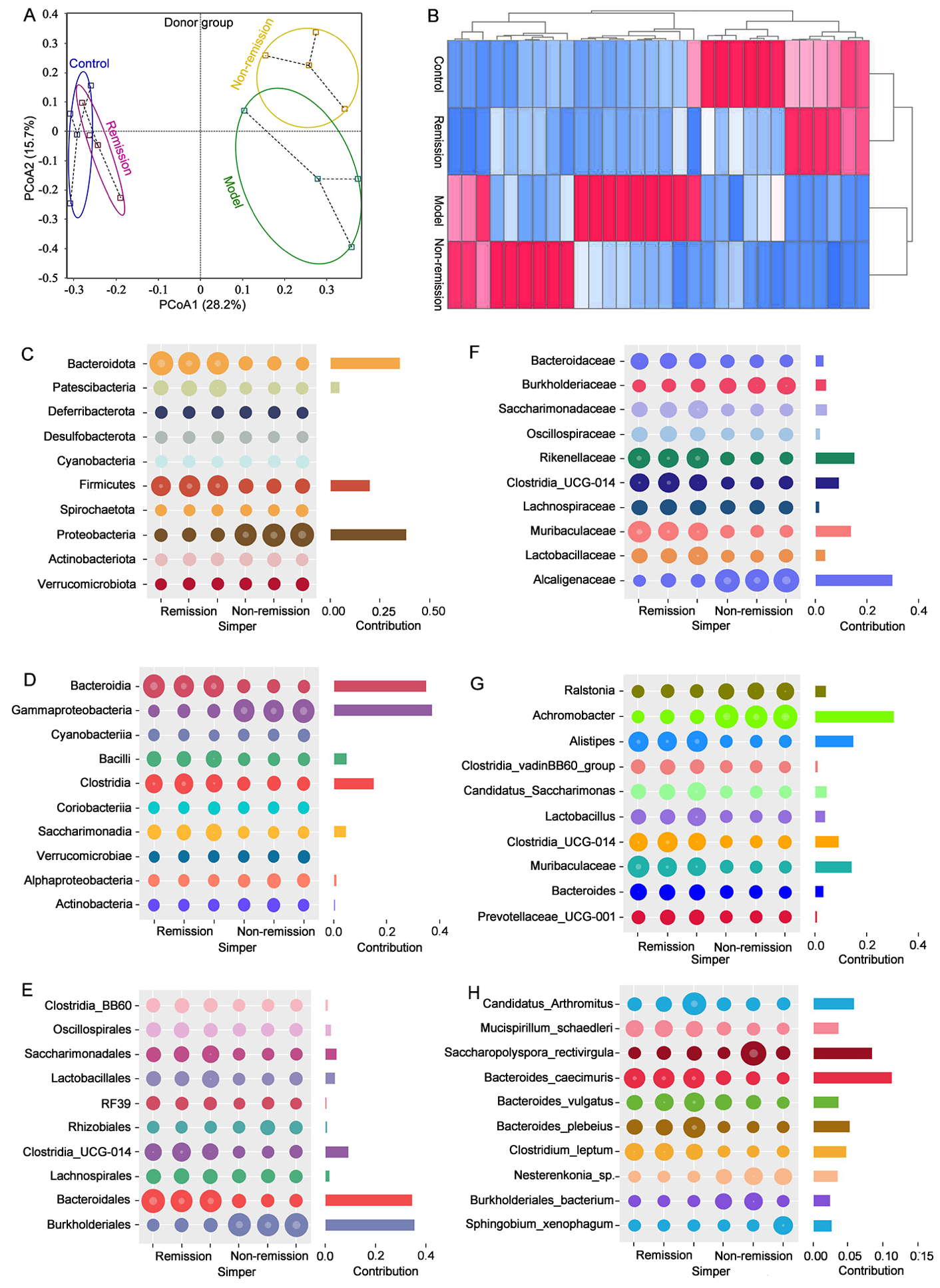


**Figure S6**

**
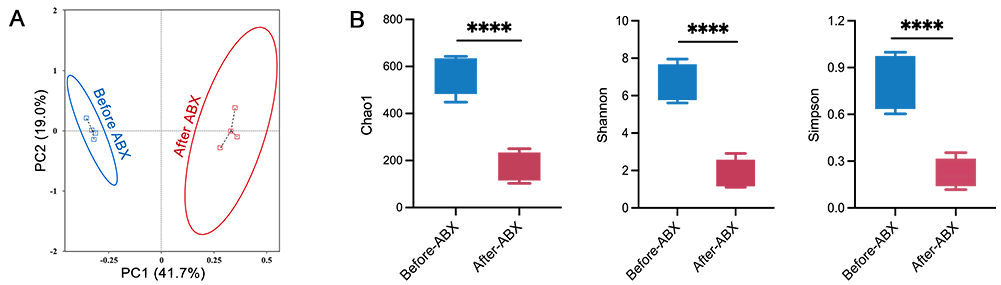
**

**Figure S7**


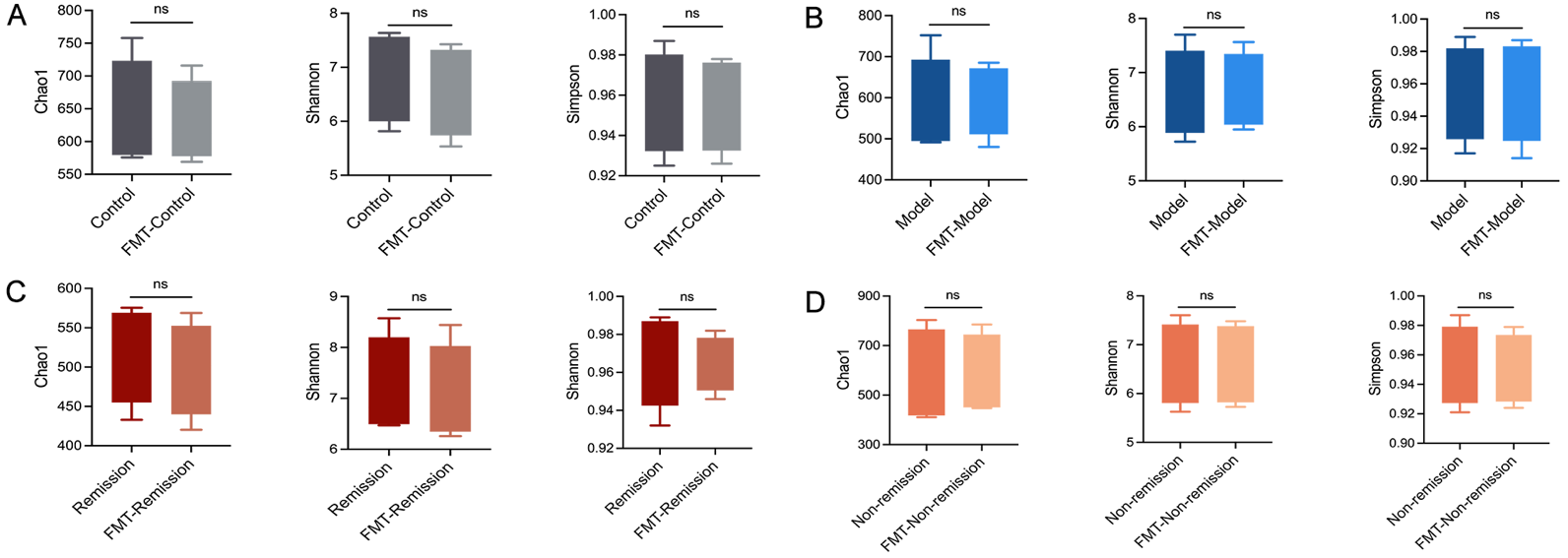


**Figure S8**


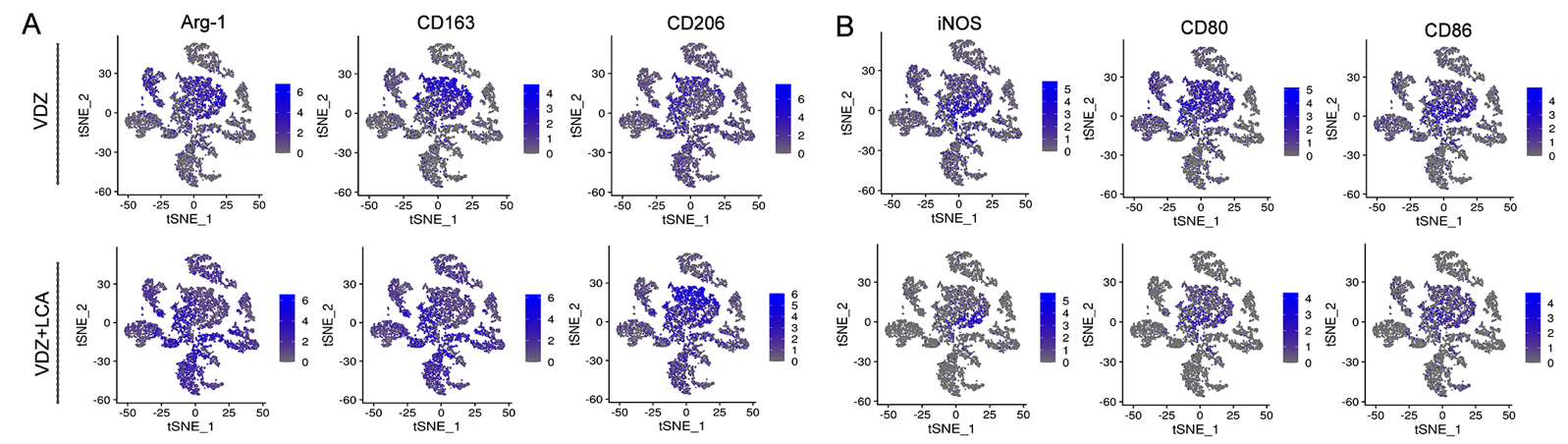


**Supplementary Tables**

**Table S1. The characteristics of donor people**

| **Health control (n=10)** | |  | **CD Patients without VDZ (n=10)** | | | | |  | | **VDZ-treated patients achieving remission (n=10)** | | | | | | |  | | **VDZ-treated patients achieving non-remission (n=10)** | | | | | | |
| --- | --- | --- | --- | --- | --- | --- | --- | --- | --- | --- | --- | --- | --- | --- | --- | --- | --- | --- | --- | --- | --- | --- | --- | --- | --- |
| **Sex** | **Age** |  | **Sex** | **Age** | | | **Disease activity at week 0** | |  | | **Sex** | **Age** | | **Disease activity at week 0** | | **Disease activity at week 14** | |  | | **Sex** | **Age** | | **Disease activity at week 0** | **Disease activity at week 14** | |
| Female | 25 |  | Female | | 27 | 11 | |  | | Female | | 24 | 13 | | 4 | | |  | | Female | 28 | 12 | | | 7 |
| Female | 31 |  | Female | | 35 | 10 | |  | | Female | | 29 | 11 | | 3 | | |  | | Female | 33 | 10 | | | 6 |
| Female | 33 |  | Female | | 41 | 11 | |  | | Female | | 33 | 10 | | 2 | | |  | | Female | 39 | 13 | | | 7 |
| Female | 37 |  | Female | | 47 | 9 | |  | | Female | | 46 | 12 | | 3 | | |  | | Female | 47 | 9 | | | 5 |
| Female | 48 |  | Male | | 31 | 12 | |  | | Female | | 55 | 10 | | 3 | | |  | | Male | 31 | 10 | | | 6 |
| Female | 52 |  | Male | | 38 | 11 | |  | | Male | | 32 | 9 | | 2 | | |  | | Male | 38 | 13 | | | 7 |
| Male | 26 |  | Male | | 42 | 12 | |  | | Male | | 40 | 11 | | 3 | | |  | | Male | 42 | 11 | | | 6 |
| Male | 30 |  | Male | | 46 | 13 | |  | | Male | | 49 | 12 | | 4 | | |  | | Male | 46 | 10 | | | 5 |
| Male | 39 |  | Male | | 51 | 10 | |  | | Male | | 50 | 11 | | 2 | | |  | | Male | 50 | 12 | | | 7 |
| Male | 43 |  | Male | | 57 | 12 | |  | | Male | | 53 | 12 | | 3 | | |  | | Male | 53 | 10 | | | 5 |

| **Table S2. Primers for reverse transcription-polymerase chain reaction analysis** | | |
| --- | --- | --- |
| **Genes** | **Direction** | **Sequences** |
| *Zo-1* | Forward | 5’-TGCAATTCCAAATCCAAACC-3’ |
|  | Reverse | 5’-AGAGACAAGATGTCCGCCAG-3’ |
| *Claudin-1* | Forward | 5’-TTAGTGGCCACAGCATGGTA-3’ |
|  | Reverse | 5’-GAAGGTGTTGGCTTGGGATA-3’ |
| *Claudin-4* | Forward | 5’-GGAGGGCCTCTGGATGAACT-3’ |
|  | Reverse | 5’-GATGCTGATGACCATAAGGGC-3’ |
| *Occludin* | Forward | 5’-TTGAAAGTCCACCTCCTTACAGA-3’ |
|  | Reverse | 5’-CCGGATAAAAAGAGTACGCTGG-3’ |
| *TNF-α* | Forward | 5’-TCCAGGCGGTGCTTGTTCC-3’ |
|  | Reverse | 5’-TGGGCTACAGGCTTGTCACTC-3’ |
| *IFN-γ* | Forward | 5’-TTCAGCTCTGCATCGTTTTGG-3’ |
|  | Reverse | 5’-TTTTCTGTCACTCTCCTCTTTCC-3’ |
| *IL-6* | Forward | 5’-TTCGGTCCAGTTGCCTTCTCC-3’ |
|  | Reverse | 5’-TTCTGAAGAGGTGAGTGGCTGTC -3’ |
| *IL-10* | Forward | 5’-CTTGCTGGAGGACTTTAAGGGTTAC-3’ |
|  | Reverse | 5’-CTTGATGTCTGGGTCTTGGTTCTC-3’ |
| *FXR* | Forward | 5’-GCAGCCTGAAGAGTGGTACT-3’ |
|  | Reverse | 5’-CAACACACAGCTCATCCCCT-3’ |
| *TGR5* | Forward | 5’-CACTGTTGTCCCTCCTCTCC-3’ |
|  | Reverse | 5’-ACACTGCTTTGGCTGCTTG-3’ |
| *Arg-1* | Forward | 5’-CCTTTCTCAAAAGGACAGCCTC-3’ |
|  | Reverse | 5’-CAGACCGTGGGTTCTTCACA-3’ |
| *iNOS* | Forward | 5’-TCTAGTGAAGCAAAGCCCAACA-3’ |
|  | Reverse | 5’-CTCTCCACTGCCCCAGTTTT-3’ |
| *GAPDH* | Forward | 5’-CTCCTGGATTTGGATTTGGA-3’ |
|  | Reverse | 5’-CTGTCCTTCAGCTGGTCCTC-3’ |
